# N-Terminal Ile-Orn- and Trp-Orn-Motif Repeats Enhance Membrane Interaction and Increase the Antimicrobial Activity of Apidaecins against *Pseudomonas aeruginosa*

**DOI:** 10.3389/fcell.2016.00039

**Published:** 2016-05-10

**Authors:** Martina E. C. Bluhm, Viktoria A. F. Schneider, Ingo Schäfer, Stefania Piantavigna, Tina Goldbach, Daniel Knappe, Peter Seibel, Lisandra L. Martin, Edwin J. A. Veldhuizen, Ralf Hoffmann

**Affiliations:** ^1^Faculty of Chemistry and Mineralogy, Institute of Bioanalytical Chemistry, Universität LeipzigLeipzig, Germany; ^2^Center for Biotechnology and Biomedicine, Universität LeipzigLeipzig, Germany; ^3^Division of Molecular Host Defence, Department of Infectious Diseases and Immunology, Faculty of Veterinary Medicine, Utrecht UniversityUtrecht, Netherlands; ^4^Molecular Cell Therapy, Faculty of Medicine, Universität LeipzigLeipzig, Germany; ^5^School of Chemistry, Monash UniversityClayton, VIC, Australia

**Keywords:** antibiotic, apidaecin, cell uptake, confocal fluorescence microscopy, proline-rich antimicrobial peptide (PrAMP), quartz crystal microbalance (QCM), transmission electron microscopy (TEM)

## Abstract

The Gram-negative bacterium *Pseudomonas aeruginosa* is a life-threatening nosocomial pathogen due to its generally low susceptibility toward antibiotics. Furthermore, many strains have acquired resistance mechanisms requiring new antimicrobials with novel mechanisms to enhance treatment options. Proline-rich antimicrobial peptides, such as the apidaecin analog Api137, are highly efficient against various *Enterobacteriaceae* infections in mice, but less active against *P. aeruginosa in vitro*. Here, we extended our recent work by optimizing lead peptides Api755 (gu-OIORPVYOPRPRPPHPRL-OH; gu = *N*,*N*,*N*′,*N*′-tetramethylguanidino, O = L-ornithine) and Api760 (gu-OWORPVYOPRPRPPHPRL-OH) by incorporation of Ile-Orn- and Trp-Orn-motifs, respectively. Api795 (gu-O(IO)_2_RPVYOPRPRPPHPRL-OH) and Api794 (gu-O(WO)_3_RPVYOPRPRPPHPRL-OH) were highly active against *P. aeruginosa* with minimal inhibitory concentrations of 8–16 and 8–32 μg/mL against *Escherichia coli* and *Klebsiella pneumoniae*. Assessed using a quartz crystal microbalance, these peptides inserted into a membrane layer and the surface activity increased gradually from Api137, over Api795, to Api794. This mode of action was confirmed by transmission electron microscopy indicating some membrane damage only at the high peptide concentrations. Api794 and Api795 were highly stable against serum proteases (half-life times >5 h) and non-hemolytic to human erythrocytes at peptide concentrations of 0.6 g/L. At this concentration, Api795 reduced the cell viability of HeLa cells only slightly, whereas the IC_50_ of Api794 was 0.23 ± 0.09 g/L. Confocal fluorescence microscopy revealed no colocalization of 5(6)-carboxyfluorescein-labeled Api794 or Api795 with the mitochondria, excluding interactions with the mitochondrial membrane. Interestingly, Api795 was localized in endosomes, whereas Api794 was present in endosomes and the cytosol. This was verified using flow cytometry showing a 50% higher uptake of Api794 in HeLa cells compared with Api795. The uptake was reduced for both peptides by 50 and 80%, respectively, after inhibiting endocytotic uptake with dynasore. In summary, Api794 and Api795 were highly active against *P. aeruginosa in vitro*. Both peptides passed across the bacterial membrane efficiently, most likely then disturbing the ribosome assembly, and resulting in further intracellular damage. Api795 with its IOIO-motif, which was particularly active and only slightly toxic *in vitro*, appears to represent a promising third generation lead compound for the development of novel antibiotics against *P. aeruginosa*.

## Introduction

Numerous classes of antibiotics developed and clinically approved in the last century have rescued millions of people from life-threatening infections. However, initial enthusiasm that bacterial pathogens are conquered, disappeared within two decades due to an increased antibiotic resistance. Bacterial resistance mechanisms are very diverse and especially pronounced in *Pseudomonas aeruginosa*. This opportunistic Gram-negative non-fermenting bacterium causes severe nosocomial infections, such as pneumonia, urinary tract infections and bacteremia, and is intrinsically resistant for different reasons: The low membrane permeability (Yoshimura and Nikaido, 1982) together with efficient efflux systems, such as MexAB-OprM and MexX-MexY, are responsible for resistances against quinolones, tetracyclines, chloramphenicol, sulfamethoxazole, trimethoprim, aminoglycosides, and some β-lactams (Li et al., 1995; Köhler et al., 1996; Zhao et al., 1998; Aires et al., 1999; Masuda et al., 2000). Beta-lactam antibiotics may also fail due to an AmpC-inducible chromosomal β-lactamase (Hancock and Speert, 2000). Furthermore, horizontal gene transfer has enabled *P. aeruginosa* strains to express aminoglycoside-modifying enzymes, whereas mutations may alter the binding sites of peptide targets (Hancock and Speert, 2000; Vakulenko and Mobashery, 2003). Adaptive resistance mechanisms are transiently triggered by environmental stimuli, such as cations, biocides, polyamides, and antibiotics, and expand the intrinsic resistance (Fernández et al., 2010, 2011). In 2010, adaptive resistance against the last resort antibiotics polymyxin B and colistin was reported, which is mediated by the two-component regulatory system ParR-ParS via activation of the arnBCADTEF operon (Fernández et al., 2010), which finally decreases the negative net charge of the outer membrane thereby lowering the binding efficiency of cationic antibiotics. The arnBCADTEF operon is also activated by low magnesium concentrations and antimicrobial peptides (AMPs) indolicidin and human cathelicidin LL-37 (Gooderham et al., 2008). Alternatively, *P. aeruginosa* can secrete the virulence factor and proteolytic enzyme elastase (also called pseudolysin) to degrade AMPs like LL-37 (Schmidtchen et al., 2002).

Clearly, there is a strong medical imperative to find and evaluate novel antibiotics, and this has led to an intensive focus on AMPs in recent years. AMPs are expressed in virtually all higher organisms as part of their innate immunity (Boman, 1995). Especially promising appear proline-rich AMPs (PrAMPs), because they can cross bacterial membranes without lysis and act by inhibition of intracellular targets (Otvos, 2002; Scocchi et al., 2011; Krizsan et al., 2014, 2015a,b). Insect-derived PrAMPs are approximately 20 residues long with the proline content typically exceeding 25%. These prolines are often incorporated into a Pro-Arg-Pro-motif resulting in high proteolytic stabilities (Bulet et al., 1999). They are especially active against *Enterobacteriaceae*, where they pass across the inner membrane via a transporter before inhibiting intracellular proteins, such as chaperone DnaK and the 70S ribosome (Kragol et al., 2001; Otvos, 2002; Mattiuzzo et al., 2007; Krizsan et al., 2014, 2015a,b). Several studies have reported high efficacies of native, optimized, and designed PrAMPs in different murine mouse infection models (Noto et al., 2008; Benincasa et al., 2010; Knappe et al., 2012). For example, Api137 (gu-ONNRPVYIPRPRPPHPRL-OH, gu = *N*,*N*,*N*′,*N*′-tetramethylguanidino, O = Orn = l-ornithine), an analog of apidaecin 1b, was optimized for antibacterial activity and proteolytic stability (Berthold et al., 2013). This PrAMP is highly active against several threatening Gram-negative bacteria by inhibiting the ribosome assembly (Krizsan et al., 2015b). Api137 shows a high *in vivo* tolerance with no acute toxic effects observed for four intraperitoneal injections of 80 mg/kg per day, while it is highly efficient in mouse infection models with *Escherichia coli* ATCC25922 providing 100% survival rates, even at low doses of only 0.6 mg/kg (unpublished data).

Recently, this lead-peptide was optimized to enhance its activity against *P. aeruginosa* in full strength media using a structure-activity relationship (SAR) study (Bluhm et al., 2015). Among several interesting peptide analogs, Api755 (gu-OIORPVYOPRPRPPHPRL-OH) and Api760 (gu-OWORPVYOPRPRPPHPRL-OH) were particularly active. Here, we report further N-terminal modifications by substituting the Asn-Asn motif in Api137 by up to three Ile-Orn- and Trp-Orn-motifs. The new PrAMPs were evaluated with respect to minimal inhibitory concentrations (MICs) against *P. aeruginosa, E. coli*, and *Klebsiella pneumoniae*, activity toward bacterial membranes assessed using quartz crystal microbalance (QCM) and transmission electron microscopy (TEM), and *in vitro* tolerance and uptake by mammalian cells investigated using confocal laser scanning microscopy and flow cytometry.

## Materials and methods

Materials were obtained from the following manufacturers: Applichem GmbH (Darmstadt, Germany): Hoechst 33342 (≥98%) and Tris ultrapure (≥99.9%); Avanti Polar Lipids (Alabaster, USA): 1,2-Dimyristoyl-sn-glycero-3-phosphocholine (DMPC) and 1,2-dimyristoyl-sn-glycero-3-phospho-rac-(1-glycerol) (sodium salt) (DMPG). Biosolve BV (Valkenswaard, Netherlands): dimethylformamide (DMF, peptide synthesis grade), dichloromethane (DCM, synthesis grade), and piperidine (synthesis grade); Bruker Daltonics GmbH (Bremen, Germany): α-cyano-4-hydroxycinnamic acid (CHCA); Carl Roth (Karlsruhe, Germany): di-potassium phosphate (≥99%), ethanol (HPLC grade), methanol (≥99%), sodium dodecyl sulfate (SDS, ≥99.5%), trichloroacetic acid (TCA ≥99%), and trifluoroacetic acid for peptide synthesis (≥99.9%); Gibco (Darmstadt, Germany): phosphate buffered saline (PBS, pH 7.4), Dulbecco's modified Eagle's medium/Ham's F-12 medium (DMEM/F-12 (1:1); Penicillin-Streptomycin (10,000 U/mL) and fetal bovine serum (FBS, qualified, heat inactivated, E.U.-approved, South America Origin); eBioscience (San Diego, USA): eFluor660; Electron Microscopy Sciences (EMS, Hatfield, USA): osmium tetroxide and uranylacetate; Greiner Bio-One GmbH (Frickenhausen, Germany): 48-well polystyrene (PS), 96-well polypropylene (PP) or PS, and 384-well PS microtiter plates; ibidi GmbH (Martinsried, Germany): μ-Slide 8 well ibiTreat; Iris Biotech (Marktredwitz, Germany): Leu-Wang resin; Life Technologies (Carlsbad, USA): MitoTracker red CMXRos, Merck (Darmstadt, Germany): calcium chloride (CaCl_2_), magnesium chloride (MgCl_2_), potassium hexacyanoferrate(II) trihydrate (K_4_Fe(CN)_6_ x3H_2_O) and *P. aeruginosa* Elastase; MultiSynTech GmbH (Witten, Germany) or Iris Biotech (Marktredwitz, Germany): all 9-fluorenylmethoxycarbonyl- (Fmoc) protected amino acids and *N*,*N*,*N*′,*N*′-tetramethyl-O-(1H-benzotriazol-1-yl)uronium hexafluorophosphate (HBTU); PAA Laboratories GmbH: mouse serum; Phenomenex Inc. (Torrance, CA, USA): Jupiter C_18_-columns [internal diameter (ID)]: 10 mm and length: 250 mm or ID: 2 mm and length: 150 mm, particle size: 5 μm, pore size: 30 nm); Polysciences (Eppelheim, Germany): glutaraldehyde; Sigma-Aldrich GmbH (Taufkirchen, Germany and Zwijndrecht, The Netherlands): 1,2-ethandithiole (≥98%), acetic anhydride, 5(6)-carboxyfluorescein (Cf, ≥95%), m-cresole (99%), thioanisole (≥99%), *N,N*′-diisopropylcarbodiimide (DIC, >98% by GC), *N,N*-diisopropylethylamine (DIPEA), hydrochloric acid (HCl, p. a.), dynasore hydrate, 1-hydroxy-benzotriazole (HOBt, >98%), low-melting point agarose, *N*-methylmorpholine (NMM, >95% GC), Mueller-Hinton broth (MHB), β-nicotinamide adeninedinucleotide reduced disodium salt hydrate (NADH), paraformaldehyde (95%), potassium phosphate monobasic (≥99.5%), TFA (UV-grade for HPLC), sodium cacodylate, sodium hydroxide (≥90%) sodium pyruvate (≥99%), methylthiazolyldiphenyl-tetrazolium bromide (MTT, ≥98%), triisopropylsilane (TIS, 98%), triton X-100, and tryptic soy broth (TSB). Sigma-Aldrich (Castle Hill, Australia): cholesterol and chloroform (≥99.8%).

### Peptide synthesis

Peptides were synthesized using Fmoc/^*t*^Bu-strategy and *in situ* DIC/HOBT activation (25-μmol scale). Side chains of trifunctional amino acids were protected with 2,2,4,6,7-pentamethyl-2,3-dihydrobenzofuran-5-sulfonyl for Arg, *tert*-butyl for Asp, Glu, Ser, Thr, and Tyr, *tert*-butyloxycarbonyl for Lys, Trp, and Orn, and trityl for Cys, His, Asn, and Gln. N-terminal residues Ile2 and Orn1 of Api796 were coupled manually with HBTU in the presence of DIPEA overnight. Cf-labeled peptides were obtained by incorporating Orn1 protected at the side chain with either the 4-methyltrityl (Mtt) (Api137) or the 1-(4,4-dimethyl-2,6-dioxocyclo-hexylidene)-3-methylbutyl (ivDde) (Api794, Api795). The N-terminal Fmoc-group was cleaved with piperidine and the *N*,*N*,*N*′,*N*′-tetramethylguanidino group incorporated by incubating the peptides with 10 equivalents of HBTU and NMM in DMF (0.5 mL). For Cf-labeling Mtt- or ivDde-groups were cleaved by repetitive treatments with 2% TFA in DCM or 2% hydrazine in DMF, respectively, before glycine, serine and Cf (8 eq.) were manually coupled with HBTU (8 eq.) and DIPEA (8 eq.). Peptides were cleaved with TFA containing a scavenger mixture (12.5% v/v; ethandithiole, *m*-cresol, water, and thioanisole, 1/2/2/2 v/v/v/v) and precipitated and washed with cold diethyl ether. Peptides were purified by RP-HPLC on a Jupiter C_18_-column (ID: 10 mm) using a linear aqueous acetonitrile gradient containing TFA (0.1% v/v) as ion pair reagent. Purities were judged by RP-HPLC and MALDI-QqTOF-MS (Synapt G2Si MS Waters, Eschborn, USA, Germany) (Figures S1, S2).

### Antimicrobial activities

MICs of *P. aeruginosa* strains DSM 1117 (ATCC 27853), DSM 3227 (ATCC 19429), and DSM 9644 were determined in a microdilution broth assay using 50 or 100% MHB (11.5 and 23 g/L MHB, respectively) whereas MICs of *E. coli* DSM 1103 (ATCC 25922) and *K. pneumoniae* DSM 681 (ATCC 10031) were determined in TSB (30 g/L TSB). Peptides (1 g/L in water) were serially two-fold diluted in a 96-well microtiter plate (PS, sterile, flat bottom) in 50 μL of the corresponding medium to final concentrations of 512–4 μg/mL. Bacteria were grown in nutrient broth overnight and diluted to a cell concentration of 1.5 × 10^7^ cells/mL in the corresponding medium. Cell densities were determined by using the McFarland standard as reference. An aliquot (50 μL) of the cell culture was added to each well and incubated (37°C, 24 h). The OD_595_ was determined in a SpectraMax 340PC (Molecular Devices, Sunnyvale, USA). Additionally, MIC values were also determined for high bacteria densities (5 × 10^8^ cells/mL) corresponding to the conditions used for TEM, as described above.

### Quartz crystal microbalance

Data were acquired with the Q-Sense E4 system (Q-sense, Sweden using polished, gold-coated, AT-cut quartz sensors with a fundamental frequency of ca. 5 MHz (Q-Sense, Västra Frölunda, Sweden) (Mechler et al., 2007; McCubbin et al., 2011). Briefly, sensors were cleaned with hydrogen peroxide (6% w/v) and aqueous ammonia (5.6% w/v) for 20 min (70–75°C), washed with water, and then activated with 3-mercaptopropionic acid (1 mmol/L in isopropanol) overnight. Liposomes consisting of DMPC and DMPG (molar ratio of 4:1) were deposited onto the sensors, washed with “high salt” PBS (20 mmol/L potassium phosphate, 0.1 mol/L NaCl, pH 6.9) and then with “low salt” PBS (10 mmol/L potassium phosphate, 30 mmol/L NaCl, pH 6.9). The system was equilibrated at 19.1 ± 0.1°C with “high salt” PBS, prior to the introduction of peptide solutions (2; 5; 10 and 20 μmol/L, 1 mL each in “high salt” PBS) at a flow rate of 50 μL/min over 20 min followed by an incubation, without any flow. Afterwards the “high salt” PBS was again introduced at 300 μL/min to determine if any surface materials could be removed from the sensor. Changes in the sensor frequency (Δ*f*) and dissipation (Δ*D*) were monitored for the third, fifth, seventh, and ninth harmonic using Q-Soft (Q-Sense) and analyzed using Origin 8 software.

### Transmission electron microscopy (TEM)

*P. aeruginosa* and *E. coli* were incubated at bacterial densities of 5 × 10^8^ colony-forming units (CFU)/mL (determined on an agar plate) with peptides at different concentrations (0.25 × MIC, MIC, 4 × MIC and 512 μg/mL, Table S1) for 1 h at 37°C. Peptide-bacteria mixtures were fixed (2% glutaraldehyde, 5 mmol/L CaCl_2_, 10 mmol/L MgCl_2_ in 0.1 mol/L sodium cacodylate buffer, pH 7.4) overnight at 4°C. Cells were washed (3 × 10 min), embedded in low-melting point agarose (2% v/v), and postfixed (4% (w/v) osmium tetroxide, 15% (w/v) K_4_Fe(CN)_6_ in distilled water) for 2 h at 4°C. Samples were rinsed with distilled water (5 × 10 min), incubated with aqueous uranylacetate (0.5% w/v) for 1 h at 4°C, washed with distilled water (3 × 10 min), and embedded in Epon. The hardened blocks were sectioned (50 nm) on a UCT ultramicrotome (Leica, Vienna, Austria), stained with uranyl acetate and lead citrate on an AC20 system (Leica), and visualized at a Tecnai 12 electron microscope (FEI, Eindhoven, The Netherlands) at 80 kV. Morphological effects were quantified on average for 25 cells per condition.

### Serum stability

Peptide solutions (1 g/L) were added to mouse serum to obtain final concentrations of 70 mg/L peptide and incubated under constant shaking at 37°C. After 0, 1, 2, 3, and 6 h aliquots (95 μL) were precipitated with aqueous TCA (25 μL, 15% w/v), incubated for 10 min on ice, and centrifuged (5 min, 12,000 × g, Eppendorf mini spin centrifuge). An aliquot of the supernatant (95 μL) was neutralized with sodium hydroxide (8 μL, 1 mol/L) and diluted with aqueous acetonitrile solution (3% acetonitrile, 0.1% TFA; 200 μL). Peptides were quantified by the peak areas obtained by analytical RP-HPLC on a Jupiter C_18_-column (ID: 2 mm) using a linear aqueous acetonitrile gradient with 0.1% (v/v) TFA (absorbance recorded at 214 nm). Peak areas of all time points were normalized to the initial peak area obtained after “0 min incubation” (= 100%). Experiments were carried out as triplicates in parallel and half-life times were calculated using GraphPad Prism 5.0.

### Elastase assay

A stock solution of *P. aeruginosa* elastase (0.26 U/μL) was prepared in Tris-HCl (5 mmol/L, pH 8.3) and stored at −80°C. Peptides (0.5 g/L in 5 mmol/L Tris-HCl, pH 8.3) were incubated with elastase (0.012 U/μL) at 37°C under constant shaking (450 rpm) for 0, 0.5, 2, 4, and 6 h. Aliquots (10.5 μL) were precipitated with a mixture of acetonitrile and ethanol (1:1 v/v) containing 0.1% TFA (21 μL), centrifuged, the supernatant transferred into a new tube, and dried in vacuum. Samples were dissolved in aqueous acetonitrile (3% v/v) containing TFA (0.1% v/v) and analyzed by RP-HPLC using the conditions described above. Half-life times were calculated using GraphPad Prism 5.0.

### Hemolysis assay

Human EDTA blood (S-Monovette® with potassium-EDTA Sarstedt AG, Nümbrecht, Germany) was centrifuged (3 min, 300 × g, 4°C) and blood cells were washed three times using the 10-fold volume of cold PBS. The diluted blood cell suspension (2% v/v in PBS, 50 μL) was mixed with a peptide solution (1.2 or 0.2 g/L in PBS, 50 μL) or triton X-100 (1%, v/v in PBS; control) in 96-well plates (PP, V-bottom). After incubation under constant shaking (1 h, 37°C, 300 rpm, Eppendorf Thermomixer) the suspension was centrifuged (3 min, 4°C, 1000 × g) and the OD_405_ of the supernatant was measured in a 384-well plate (PS, flat bottom, Paradigm microplate reader, Molecular Devices, Sunnyvale, USA). The hemolytic grade (HG) was calculated as [(OD_Peptide_−OD_PBS_)/(OD_Triton_−OD_PBS_)] × 100%.

### Cytotoxicity

Human embryonic kidney (HEK293) and HeLa cells were cultured in Dulbecco's modified Eagle's/Ham's F-12 medium [DMEM/F-12 (1:1)] supplemented with FBS (10% v/v), penicillin, and streptomycin (1000 units each). Primary rat cardiomyocytes (Innoprot, Elexalde Derio, Spain) were grown in medium supplemented with FBS (20% v/v), horse serum (5% v/v), L-glutamine (2 mmol/L), sodium pyruvate (2 mmol/L), MEM non-essential amino acids (Gibco, 0.1 mmol/L), penicillin, and streptomycin (1000 units each). Surfaces for cardiomyocyte growth were coated with attachment factor first. Cells (20,000/well) were seeded into a 96-well plate (PS, sterile, flat bottom), incubated for 24 h (37°C, 5% CO_2_), and washed twice with PBS (100 μl/well) prior to adding colorless medium (88 μL) and peptide solution (12 μL; 5, 2.5, 1.25, 0.75, and 0.625 g/L in PBS). Positive controls were triton X-100 (1%, v/v) and melittin (60 mg/L) as lytic peptide, whereas PBS (12%, v/v) served as negative control. After an incubation period of 24 h, supernatant (25 μL) was mixed with lactate dehydrogenase (LDH) substrate (175 μL; 0.57 mmol/L sodium pyruvate, 0.24 mmol/L NADH disodium salt, 32 mmol/L potassium dihydrogen phosphate, 66 mmol/L dipotassium phosphate) in 96-well plates (PS, sterile, flat bottom). Absorbance was recorded at 340 nm every 3 min for 30 min on the Paradigm microplate reader. Due to the total release of LDH upon triton X-100 treatment, the control contained high LDH concentrations resulting in a fast substrate turnover that was only linear for the first 6 min. Hence, the differences of the absorbances recorded initially (*t* = 0 min) and after 6 min (ΔOD) were used for all samples and normalized to the corresponding values obtained for triton X-100 at the time points using the equation [(ΔOD_Peptide_ − ΔOD_PBS_)/(ΔOD_Triton_ − ΔOD_PBS_)] × 100%.

The MTT cell viability assay relied on the same cell culture. Remaining medium was replaced by a mixture of fresh colorless medium (100 μL) and MTT (10 μL; 5 g/L in PBS) and incubated for 4 h (37°C, 5% CO_2_). A solution of sodium dodecyl sulfate (100 μL; 10%, w/v) dissolved in hydrochloric acid (10 mmol/L) was added, incubated (16 h), and the absorbance recorded at 590 nm relative to the reference at 650 nm (Paradigm microplate reader). The relative cell viability was calculated by [(OD_Peptide_ − OD_Triton_)/(OD_PBS_ − OD_Triton_)] × 100% and half-maximal inhibitory concentrations (IC_50_) with an Excel-template from cell biology protocols (www.sciencegateway.org/ protocols/cellbio/drug/hcic50.htm). For statistical analysis an unpaired *t*-test was applied using GraphPad Prism 5.0.

### Confocal laser scanning microscopy

HeLa cells (50,000 in 300 μL DMEM/F-12 medium) were seeded into μ-slides (eight wells) and incubated (23 ± 1 h) using the same conditions as described above and washed twice with PBS (300 μL/well).

#### Mitochondrial stain

Fresh colorless medium (144 μL) containing MitoTracker Red CMXRos (0.1 μmol/L) and Cf-labeled peptide (6 μL; 1 mmol/L in water) was added and incubated (6 h, 37°C). The cells were washed three times (300 μL PBS/well), fixed with formaldehyde (2% w/v in PBS, 150 μL, 15 min) at room temperature (RT), and washed twice (300 μL PBS/well) before Hoechst 33342 stain was added (2 μmol/L in PBS, 300 μL). After 30 min (RT) the supernatant was removed and fresh PBS (300 μL) added.

#### Endocytosis inhibition

Fresh colorless medium (150 μL) supplemented with either dynasore (0.2 mmol/L) dissolved in DMSO (final concentration of 0.2% v/v) or DMSO (0.2% v/v) as vehicle control was added. After 45 min (37°C) the supernatant was replaced by fresh medium supplemented with either dynasore or DMSO at the same concentrations (144 μL), Cf-labeled peptide added (6 μL, 1 mmol/L in water), and incubated (30 min, 37°C). Cells were fixed and stained with Hoechst 33342 as described above.

Cells were imaged on a TCS SP5 microscope (Leica Mikrosysteme, Wetzlar, Germany) using a HCX PL APO λ blue 63 × /1.4 OIL UV lens (number 11506192) and the LAS AF 2.6.0 software. Hoechst 33342, 5(6-)carboxyfluorescein, and MitoTracker Red CMXRos were detected using at 405 nm (diode laser), 488 nm (argon laser), and 561 nm (DPSS laser), respectively.

### Flow cytometry

HeLa cells (50,000 for endocytosis inhibition and 70,000 for uptake kinetics) suspended in DMEM/F-12 medium (300 μL; see above) were seeded into 48-well plates (PS, sterile, flat bottom) and incubated (23 ± 1 h) using the conditions described above. Cells were washed twice with PBS (300 μL/well) and fresh medium was added (144 μL).

#### Cell uptake

Peptide solutions (6 μL; 1 mmol/L in H_2_O) were added to achieve a concentration of 40 μmol/L at different time points. Cells were washed three times with PBS (300 μL/well), treated with trypsin (75 μL; 0.05% w/v trypsin, 5 min, 37°C), rinsed off the plate using cold PBS (600 μL/well), and trypsin was removed by two washing steps (400 × g, 4°C, 4 min, 600 μL PBS each). Cells were suspended in PBS (0.1 mL) containing eFluor660 (1000 × diluted in PBS) and incubated on ice in the dark. After 30 min, cells were washed with cold PBS twice, suspended in formaldehyde (100 μL; 2% w/v in PBS), incubated (15 min, RT), washed (200 μL PBS), stored in FACS buffer (200 μL; 3% FBS, 0.1% NaN_3_ in PBS) overnight, and analyzed on a FACSCalibur (Becton Dickinson, New Jersey, USA). Alternatively, cells were fixed directly without prior eFluor660 staining.

#### Endocytosis inhibition

Cell cultures were prepared and incubated in the presence of dynasore, as described for microscopy. Cells were washed, treated with trypsin, and fixed as described above, dissolved in FACS buffer, and analyzed the same day.

## Results

### Antimicrobial activity

A recent SAR study provided analogs of Api137 that were more active against *P. aeruginosa* due to an increased positive net charge and hydrophobicity, such as Api755 and Api760 that were 16 times more active in 50% MHB than Api137 (Bluhm et al., 2015). In order to increase the net charge and the hydrophobicity, we elongated the N-terminal sequence by repeating the Ile-Orn- and Trp-Orn-motifs two or three times, respectively. The MIC values of the resulting analogs Api793, Api794, Api795, and Api796 were around two-fold more active against the tested *P. aeruginosa* strains than Api755 and Api760 in 50% MHB (Table 1). In full-strength MHB, the MICs of Api793 and Api796 were 64 μg/mL (*P. aeruginosa* DSM 1117) and thus two-fold lower than for Api755 and Api760, whereas peptides Api794 and Api795 were four-fold more active (MIC = 32 μg/mL). *P. aeruginosa* DSM 3227 was two-fold more susceptible to peptides containing three repeats (Api794 and Api796) than to Api755 and Api760, whereas *P. aeruginosa* DSM 9644 was two- and eight-fold more susceptible against Api793 and Api794, respectively, than the other four analogs. Importantly, the antimicrobial activities against *E. coli* DSM 1103 were only slightly reduced to MICs of 8 μg/mL (only Api794 was two-fold less active). *K. pneumoniae* DSM 681 was most susceptible against Api795 (MIC = 8 μg/mL), whereas Api793 and Api796 were two-fold and Api794 even four-fold less active.

**Table 1:** **Peptide sequences and minimal inhibitory concentrations (MICs) of Api137 and its new analogs**.

**Code**	**Sequence**	**MIC [**μ**g/mL]**							
		***P. aeruginosa***							
		**DSM 1117**	**DSM 3227**	**DSM 9644**	**DSM 1117**	**DSM 3227**	**DSM 9644**	***E. coli* ATCC 25922**	***K. pneumoniae* DSM 681**
		**50% MHB**			**100% MHB**			**TSB**	
Api137	guONNRPVYIPRPRPPHPRL	256	>256	256	>256	>256	>256	2	16
Api755	guOIORPVYOPRPRPPHPRL	16	64	16	128	256	128	8	8
Api760	guOWORPVYOPRPRPPHPRL	16	32	8	128	256	128	8	8
Api793	guO(WO)_2_RPVYOPRPRPPHPRL	16	16	8	64	256	64	8	16
Api794	guO(WO)_3_RPVYOPRPRPPHPRL	16	16	16	32	128	16	16	32
Api795	guO(IO)_2_RPVYOPRPRPPHPRL	8	16	8	32	256	128	8	8
Api796	GuO(IO)_3_RPVYOPRPRPPHPRL	8	16	16	64	128	128	8	16

*Activities were determined against three strains of P. aeruginosa in 50% and 100% MHB and one strain of E. coli and K. pneumoniae in TSB*.

### Cytotoxicity

**Figure 1:**
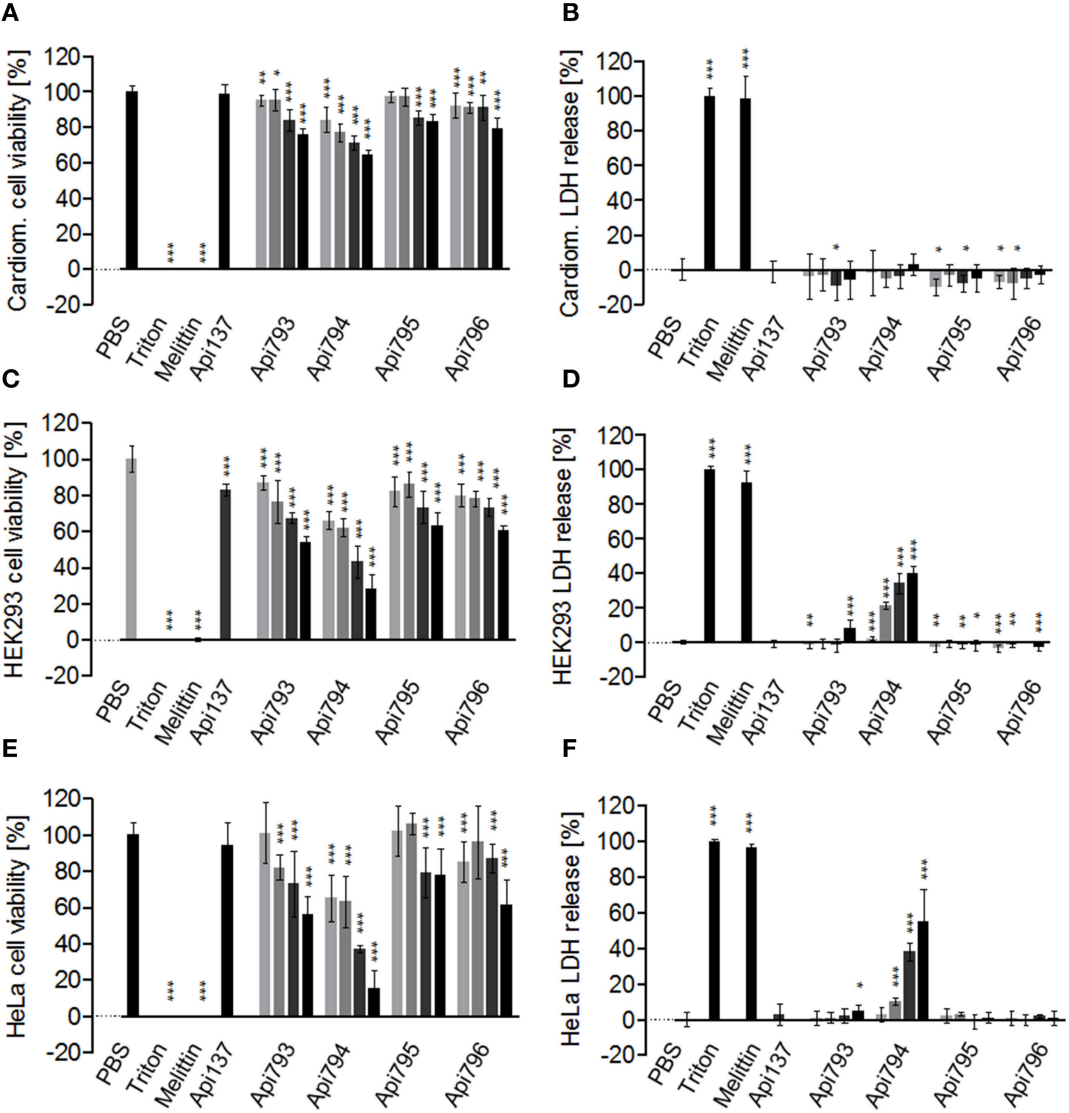
**Cytotoxic effects of Api137 studied for rat cardiomyocytes (A,B), HEK293 (C,D), and HeLa (E,F) cell lines**. Cells were incubated with peptide concentrations of 0.075, 0.15, 0.3, and 0.6 g/L (light gray to black) for 24 h. Cell viability was determined in an MTT assay and cell membrane permeabilization in a LDH assay. PBS (0% LDH release and 100% cell viability, respectively) and 1% triton X100 (100% LDH release and 0% cell viability, respectively) were used as controls. The lytic peptide melittin (0.06 g/L) served as positive control. For statistical analysis, the unpaired *t*-test was used. ^***^P ≤ 0.001; ^**^P ≤ 0.01; ^*^P ≤ 0.05.

Hemolytic grades of all four optimized peptides Api793 to Api796 were below 2%, which was at the background level. This clearly indicates that all peptides were non-hemolytic to human erythrocytes even at high concentrations of 0.6 g/L (Table 2). All four new apidaecin analogs affected the cell viability of rat cardiomyocytes (Table 2, Figure 1A). After an incubation period of 24 h, the highest concentration tested (0.6 g/L) for Api794 and Api795 decreased the cell viability to 64 ± 3% and to 83 ± 4%, respectively. For all peptides, the IC_50_ values were much above the tested concentration range and could thus not be calculated. This was consistent with the LDH assay indicating that cells did not release LDH at detectable quantities when incubated with any apidaecin analog (Figure 1B). HEK293 cells were more susceptible to apidaecin analogs including Api137 with a cell viability of 83 ± 3% at the highest concentration (Figure 1C) (Berthold et al., 2013). Cell viabilities decreased to around 60% for Api795 and Api796 containing Ile-Orn-motifs providing IC_50_ values of >0.6 g/L. Api793 and Api794 containing two and three Trp-Orn-motifs, respectively, were more toxic with IC_50_ values of 0.64 ± 0.05 g/L and 0.28 ± 0.03 g/L. Consistently, Api794 added at a concentration of 0.075 g/L released LDH already at a rate of 2 ± 1% relative to triton X-100, which increased to 40 ± 4% at the highest probed concentration, whereas Api793 induced a slight release of LDH (8 ± 5%) at the highest peptide concentration. In contrast, a LDH release was not detected for Api137, Api795, and Api796 (Figure 1D).

**Table 2:** **Hemolytic grades against human erythrocytes, cytotoxicity (IC_50_) against rat cardiomyocytes, HEK293 cells, and HeLa cells, and half-life times in mouse serum determined for Api137 and the four new apidaecin analogs**.

	**Hemolytic grade [%]**		**IC**_**50**_ **on cell viability [g/L]**			**Half-life time mouse serum (min)**
	**0.1 g/L**	**0.6 g/L**	**Rat cardiomyocytes**	**HEK293**	**HeLa**	
Api137^*^	0.4 ± 0.5	1.0 ± 0.2	>0.6	>0.6	>0.6	345
Api793	1.3 ± 0.4	1.6 ± 0.5	>0.6	0.64 ± 0.05	0.64 ± 0.15	246
Api794	1.7 ± 0.2	1.9 ± 0.3	>0.6	0.28 ± 0.03	0.23 ± 0.09	311
Api795	−0.8 ± 0.4	0.0 ± 0.7	>0.6	>0.6	>0.6	354
Api796	−0.8 ± 0.2	−0.1 ± 0.8	>0.6	>0.6	>0.6	249

* *Data for Api137 were already published (Berthold et al., 2013; Bluhm et al., 2015)*.

In agreement with the literature (Berthold et al., 2013; Bluhm et al., 2015), Api137 did not affect the cell viability of HeLa cells (Figure 1E), whereas Api795 reduced the cell viability to 78 ± 14% and Api796 to 61 ± 14%. Again, Api793 and Api794 were the most cytotoxic peptides with IC_50_ values of 0.64 ± 0.15 g/L and 0.23 ± 0.09 g/L, respectively. The LDH assay indicated that Api137, Api795, and Api796 did not release LDH, while Api793 had a small effect (5 ± 3%) at a concentration of 0.6 g/L (Figure 1F). Again, Api794 released most LDH with an EC_50_ of 0.56 ± 0.16 g/L.

### QCM studies

**Figure 2:**
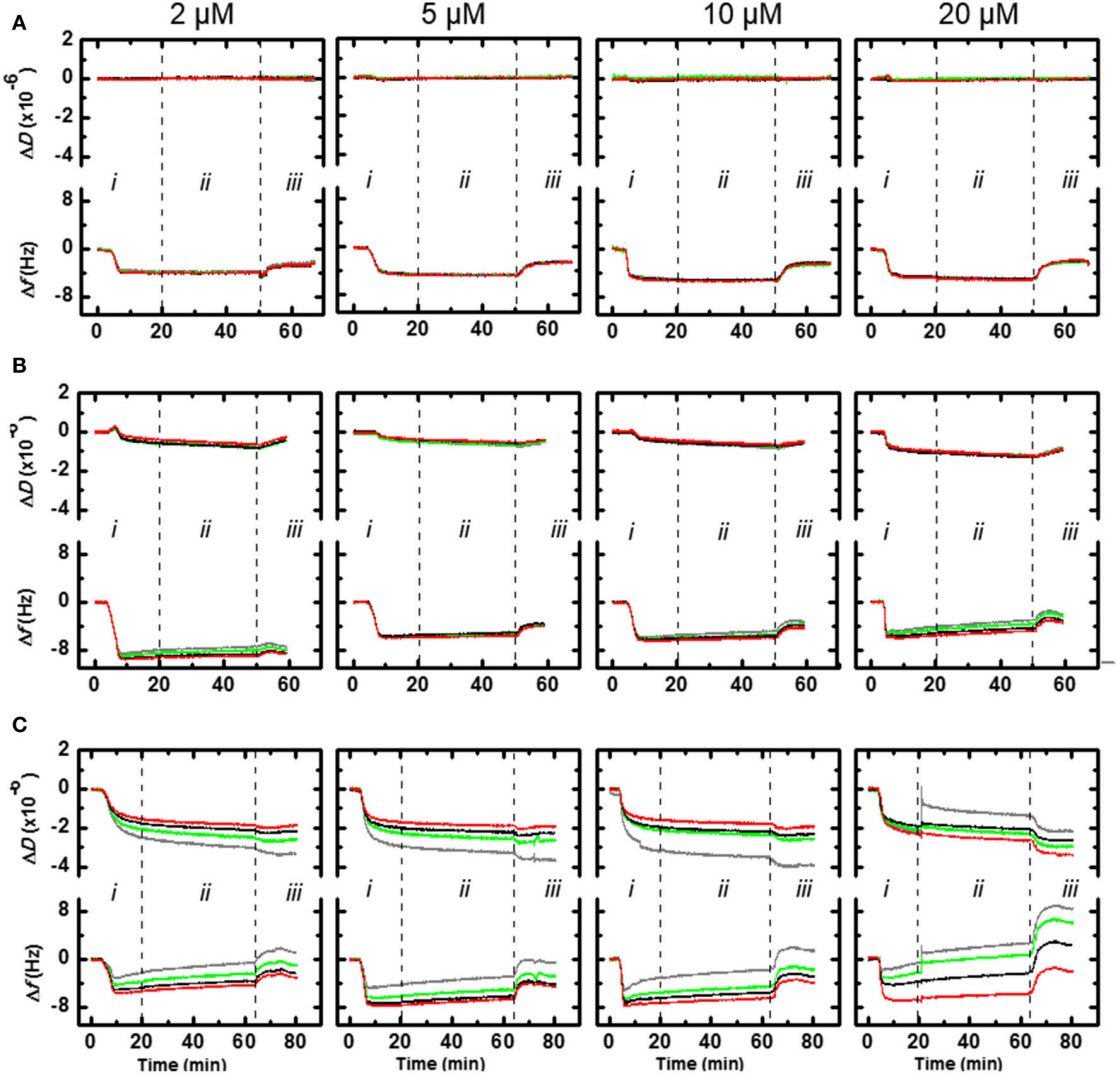
**QCM frequency and dissipation for Api137 (A), Api795 (B), and Api794 (C) acting on surfaces coated with DMPC:DMPG (4:1) as bacterial membrane mimics**. Shown are normalized data as Δ*D-t* and Δ*f* -t graphs of 3rd (gray), 5th (green), 7th (black), and 9th (red) harmonics. Peptides were loaded into a flow cell (period *i*), incubated (no flow) (period *ii*), and PBS buffer introduced into the chamber (period *iii*).

Based on the high activity against *P. aeruginosa*, Api794 and Api795 appeared the most promising lead compounds and thus were further evaluated for the interaction with bacterial membranes. The QCM sensor coated with a bacterial mimetic lipid mixture (DMPC:DMPG, molar ratio of 4:1) showed Δ*f* values that decreased over time when peptide Api137 inserted (binding) to the membrane (Figure 2A, *i*). As the frequency changed similarly for all four harmonics assessed in parallel (3rd, 5th, 7th, 9th) the insertion of the peptide was most likely trans-membrane. For Api137 the magnitude of Δ*f* (4–5 Hz) was independent of concentration over the range 2–20 μmol/L. No change in dissipation (Δ*D*), a measure of the viscoelasticity of the membrane, was observed at the concentrations examined indicating that the membrane structure was not affected by Api137 during the incubation period *ii*. During period *iii* of the experiment, washing with PBS buffer solution removed approximately half of peptide Api137 (~2 Hz) from the membrane layer (Figure 2A). Once again, no change in dissipation was detected during this period. Qualitatively the (IO)_2_ peptide (Api795) also showed a transmembrane insertion into the DMPC:DMPG membrane layer; Δ*f* = 9 Hz at 2 μmol/L and 6 Hz for 5, 10, and 20 μmol/L (Figure 2B, *i*). This variation was not significant but the 2 μmol/L data appeared to be greater due to a thicker membrane layer. The 5–20 μmol/L data showed a very slight spreading of the harmonics as the concentration increases indicating that there was a small change in the organization of the peptide-membrane layer during incubation period *ii*. This was also reflected by a small decrease in Δ*D*-t as the concentration of Api795 increases. This corresponds to some membrane restructuring through incorporation of the peptide, generating a more rigid layer. Upon introduction of the PBS buffer to the Api795-loaded membrane the frequency loss was ~1–1.5 Hz and the dissipation change was negligible. The QCM data for Api794 showed the maximum Δ*f* = 6–7 Hz for the higher (9th) vs. lower (3rd) harmonics for all the concentrations (Figure 2C, *i*). This data was consistent with the other peptides in-so-far as the Api794 peptide inserted in a transmembrane manner; however, the influence of the (WO)_3_-motif supported an organization of the peptide that resulted in more surface activity. This effect was more apparent at the highest concentration (20 μmol/L) where the harmonics monitored during the incubation period *ii* spread (Figure 2C). The change in dissipation for 2–10 μmol/L also showed a differential response across the four harmonics examined. This effect was greatest for the 3rd harmonic, which reflects the lipid surface and less so, for the 9th harmonic, which probed deeper within the lipid layer. This was consistent with the greater surface re-structuring of the membrane by the presence of tryptophan residues in the Api794.

### Transmission electron microscopy

In order to determine peptide-induced morphological changes, *P. aeruginosa* and *E. coli* were incubated with different concentrations of Api137, Api794, and Api795 based on their individual MIC values for providing a representative comparison among the peptides (Table S1). Thus, images of 25–30 bacteria were (semi) quantitatively scored for each peptide and each concentration (Tables S2, S3).

**Figure 3:**
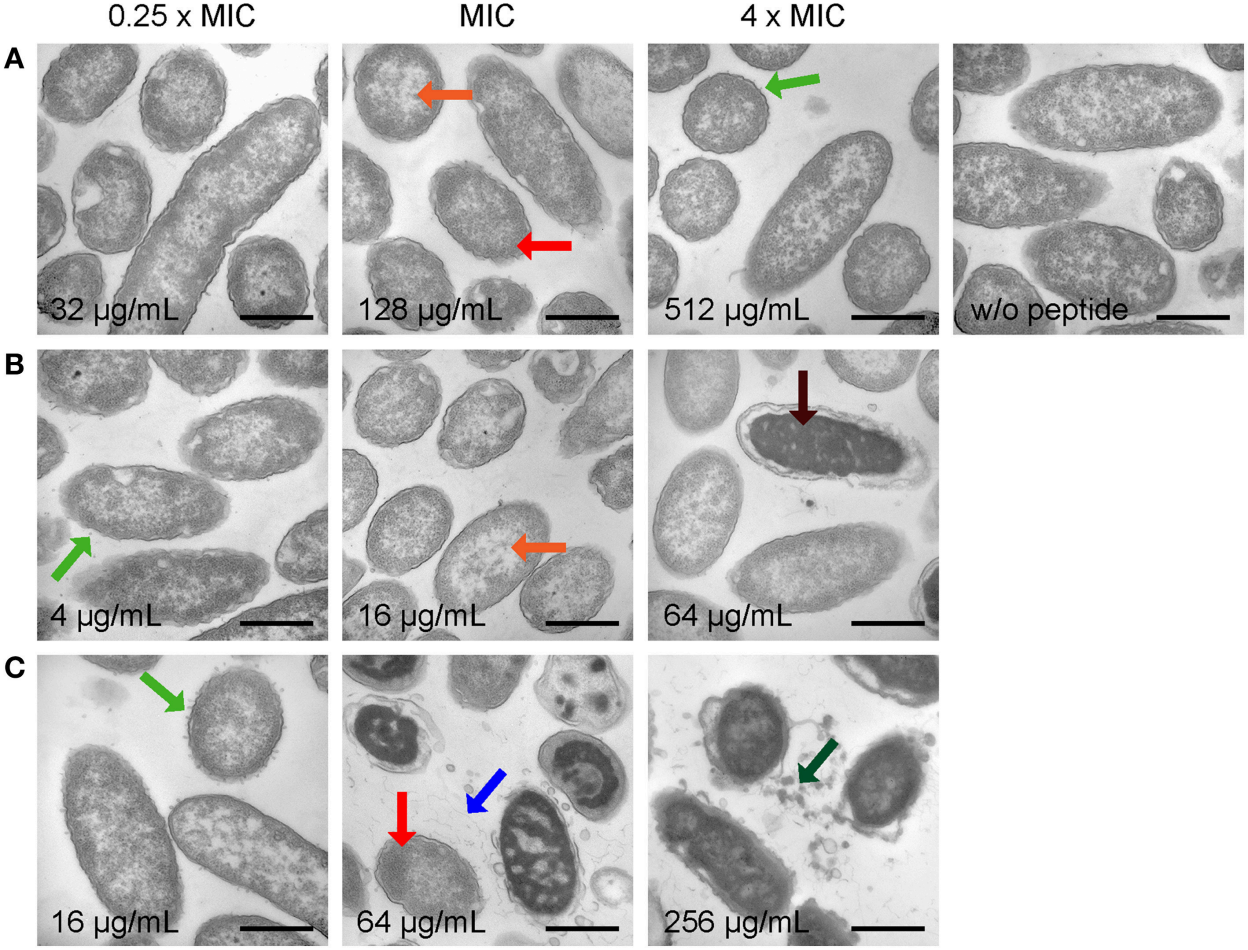
**Electron microscopy images of *P. aeruginosa* DSM 1117 incubated with Api137 (A), Api795 (B), and Api794 (C) at concentrations corresponding to 0.25 × MIC, MIC, and 4 × MIC for 1 h using cell densities of 5 × 10^8^ CFU/mL**. Black bars represent 500 nm. Morphological changes are indicated with arrows: dissociated fragments (blue), small vesicle release (green), large vesicle release (dark green), relocalized DNA (orange), slightly clustered ribosomes (red), and extremely clustered ribosomes (dark red).

Control cells of *P. aeruginosa* displayed equally distributed DNA and ribosomes (white and black areas, respectively) and intact but wrinkled membranes (Figure 3, Figure S3, both top right). Upon treatment with peptides, DNA and ribosomes relocalized. Electron dense areas containing ribosomes were clustered close to membranes while DNA-rich lighter areas appeared in the center of the bacteria (Figure 3, Figure S3). Additionally, and depending on the peptide and its concentration, up to 20 small vesicles attached outside of or just released from the bacterial membrane were observed at each cell. Larger effects on the pseudomonal membrane included larger dissociated fragments and highly deformed membranes or complete membrane ruptures. However, a complete loss of the cytoplasmic content was not observed for any of the conditions tested.

Specifically, Api137 induced some intracellular re-localization of DNA and ribosomes as well as vesicle formation, but both effects did not correlate to the concentration. Effects on the bacterial membranes or increasing amounts of dissociated fragments were not monitored, not even at 4 × MIC (Figure 3A, Figure S3A). The evidence of morphological changes observed for Api795 and Api794 correlated much clearer to the peptide concentration. At sub-MIC peptide values, only small intracellular changes were observed for both peptides (DNA/ribosomal reorganization) while the membranes were still intact (Figures 3B,C, Figures S3B,C). When treated with Api795 at 4 × MIC, membranes ruptured in 10% of the cells, which occurred even in 80% of the cells incubated with 512 μg/mL (Figure 3B, Figures S3B, S5A). Api794 already caused membrane rupture at MIC, whereas spongy-like appearances were visible at the highest probed concentrations indicative of strong distortion of the bacterial cell (Figure 3C, Figures S3C, S5A). Furthermore, Api794 strongly disrupted cell membrane fragments releasing specific dense large vesicles at the two highest concentrations distinct from the small membrane attached vesicles observed at low concentrations. Interestingly, the ruptures initiated by Api795 at 512 μg/mL and Api794 at lower concentrations were mainly observed for inner membranes indicating that peptides might have direct effects specifically on these membranes.

**Figure 4:**
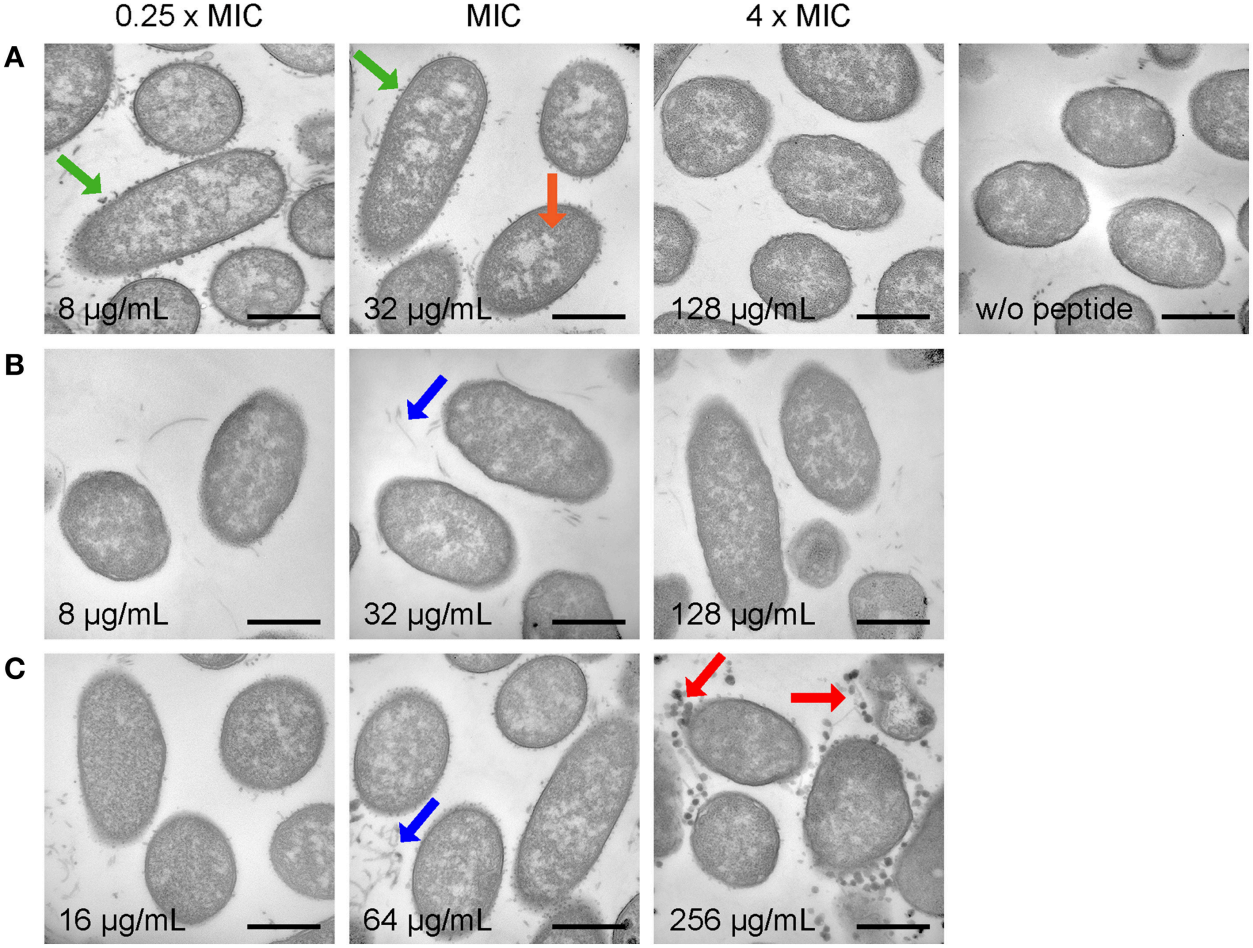
**Electron microscopy images of *E. coli* DSM 1103 incubated with Api137 (A), Api795 (B), and Api794 (C) at concentrations corresponding to 0.25 × MIC, MIC, and 4 × MIC for 1 h using cell densities of 5 × 10^8^ CFU/mL**. Black bars represent 500 nm. Morphological changes are indicated with arrows: dissociated fragments (blue), small vesicle release (green), large vesicle release (red), and relocalized DNA (orange).

Untreated *E. coli* cells showed a homogenous distribution of DNA and ribosomes with only a few short dissociated fragments in the section (Figure 4, Figure S4, both top right). A small number of these control cells appeared to have some cytoplasm retraction and wrinkled membranes, which is sometimes observed during fixation of the bacteria in our hands. Contrary to *P. aeruginosa*, all peptides triggered only mild effects on the morphology of *E. coli*, even at high concentrations of 4 × MIC. For instance, intracellular effects were limited to slight DNA and ribosome relocalization in some cells treated with Api137 at 0.25 × MIC and MIC and in cells treated with 512 μg/mL of Api795 and Api794 (Figure 4A, Figures S4A, S5B), while no membrane disruptions were observed in any of the peptide-treated samples. Interestingly, incubation of bacteria with sub-MIC of Api137 induced strong vesicle formation at the outer membrane (Figure 4A, Figure S4A), and comparable structures were seen for Api794 at its MIC and above (Figure 4C, Figure S4C). Interestingly, at 4 × MIC of Api794 larger vesicular, more filled structures not attached to the bacteria anymore were observed, while at higher concentrations of Api137 the vesicles had disappeared, making it unclear if the two types of vesicles have a common origin.

### Proteolytic stability

**Figure 5:**
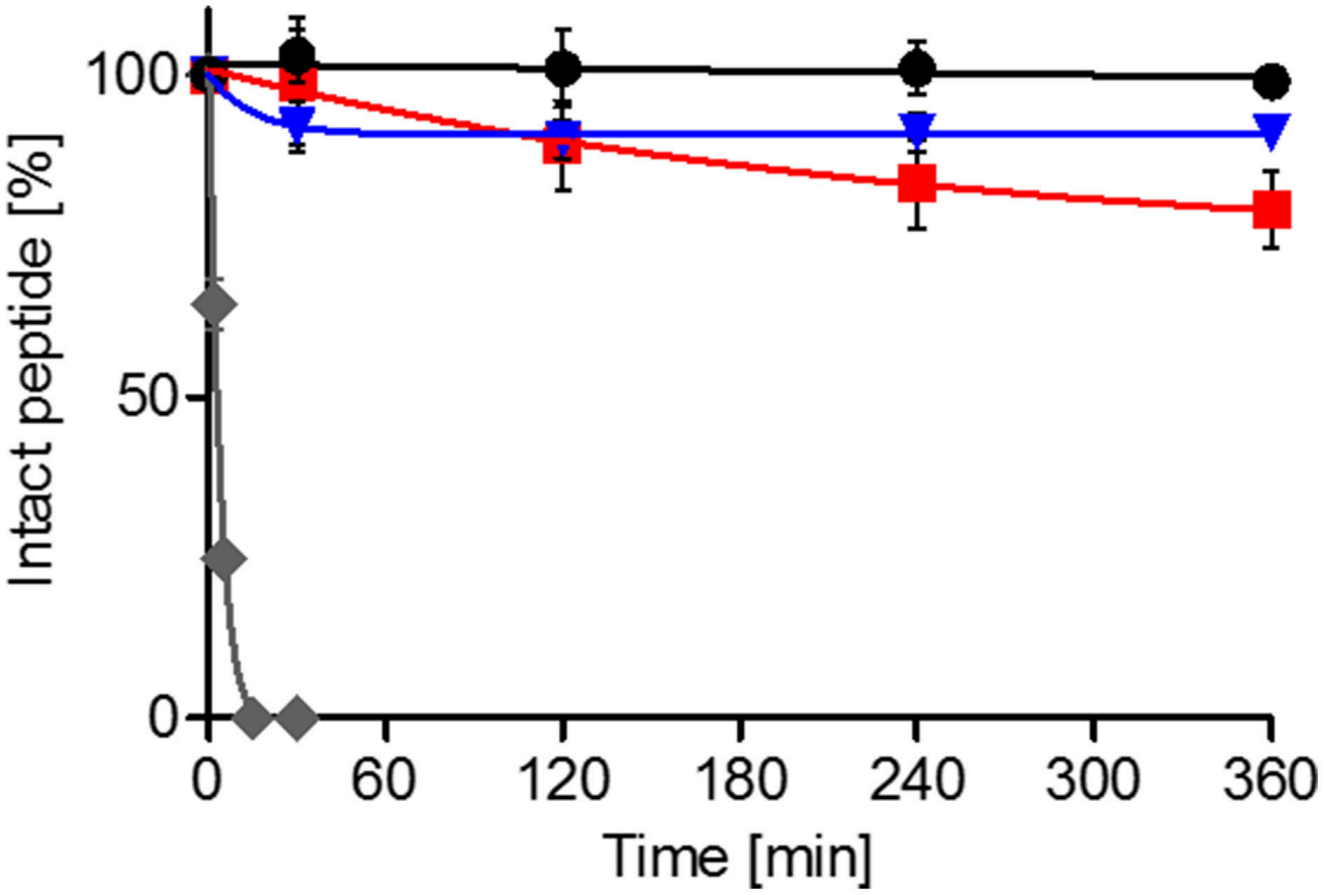
**Stability of Api137 (black), Api795 (blue), and Api794 (red) in the presence of elastase**. Peptides (0.5 g/L) were incubated with *P. aeruginosa* elastase (12 units/mL) in Tris-HCl buffer (pH 8.3) at 37°C and quantified by RP-HPLC. Peptide quantities are shown relative to the initial peak area (0 min). LL-37 (gray) was used as positive control.

Api137 is relatively stable in mouse serum with a half-life time of 345 min (Berthold et al., 2013). The new analogs Api794 and Api795 were equally stable with half-life times of 311 and 354 min (Table 2), respectively, indicating that both Ile-Orn- and Trp-Orn-motifs do neither induce new proteolytic cleavage sites nor accelerate cleavages in the C-terminal part. Besides serum protease stabilities important for systemic applications, degradation by proteases secreted by bacteria as potential resistance factors is also an important consideration for judging the therapeutic potential of AMPs (Schmidtchen et al., 2002; Sieprawska-Lupa et al., 2004; Mattiuzzo et al., 2014). In agreement with the literature *Pseudomonas* elastase degraded LL-37 with a half-life time of only 3 min (Schmidtchen et al., 2002; Nomura et al., 2014) yielding three degradation products [LL37(32-37), LL37(33-37), and LL37(1-30)], whereas Api137 was fully recovered after an incubation period of 6 h (Figure 5, Figure S6). Api794 was slowly degraded with 79 ± 6% remaining after 6 h, whereas 92 ± 4% of Api795 was detected after 30 min and for all later time points. Thus, apidaecin analogs can overcome this elastase-dependent resistance mechanism of *P. aeruginosa*.

### Uptake in HeLa cells

**Figure 6:**
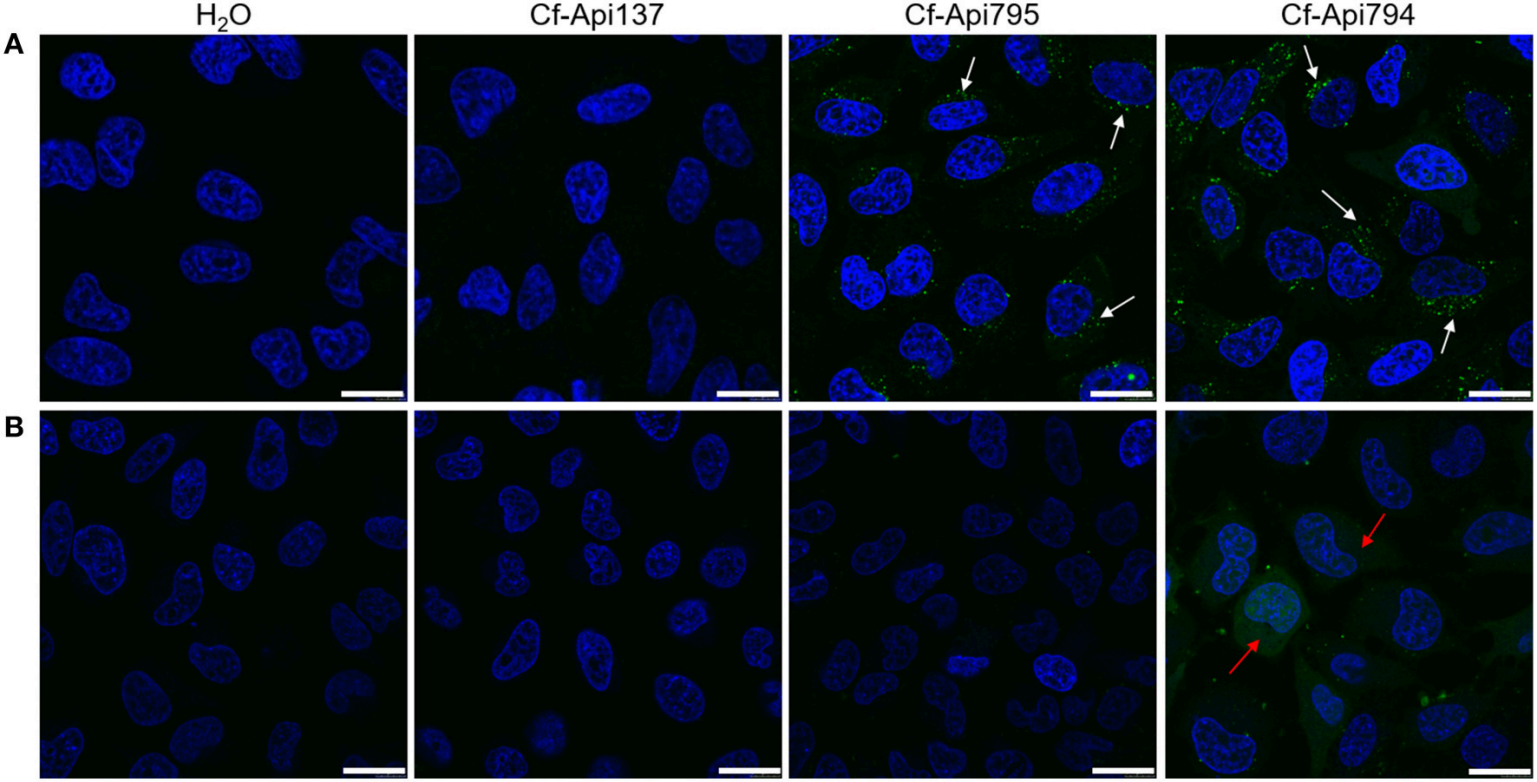
**Confocal microscopy images of HeLa cells incubated with Cf-labeled apidaecin peptides**. Cells were treated without **(A)** or with dynasore (0.2 mmol/L; **B**) for 45 min prior to peptide treatment (40 μmol/L, 30 min). The cells' nuclei were visualized with Hoechst 33324 (blue). Arrows indicate examples of areas with endosomes (white) and stained cytosol (red). Bars refer to 20 μm.

**Figure 7:**
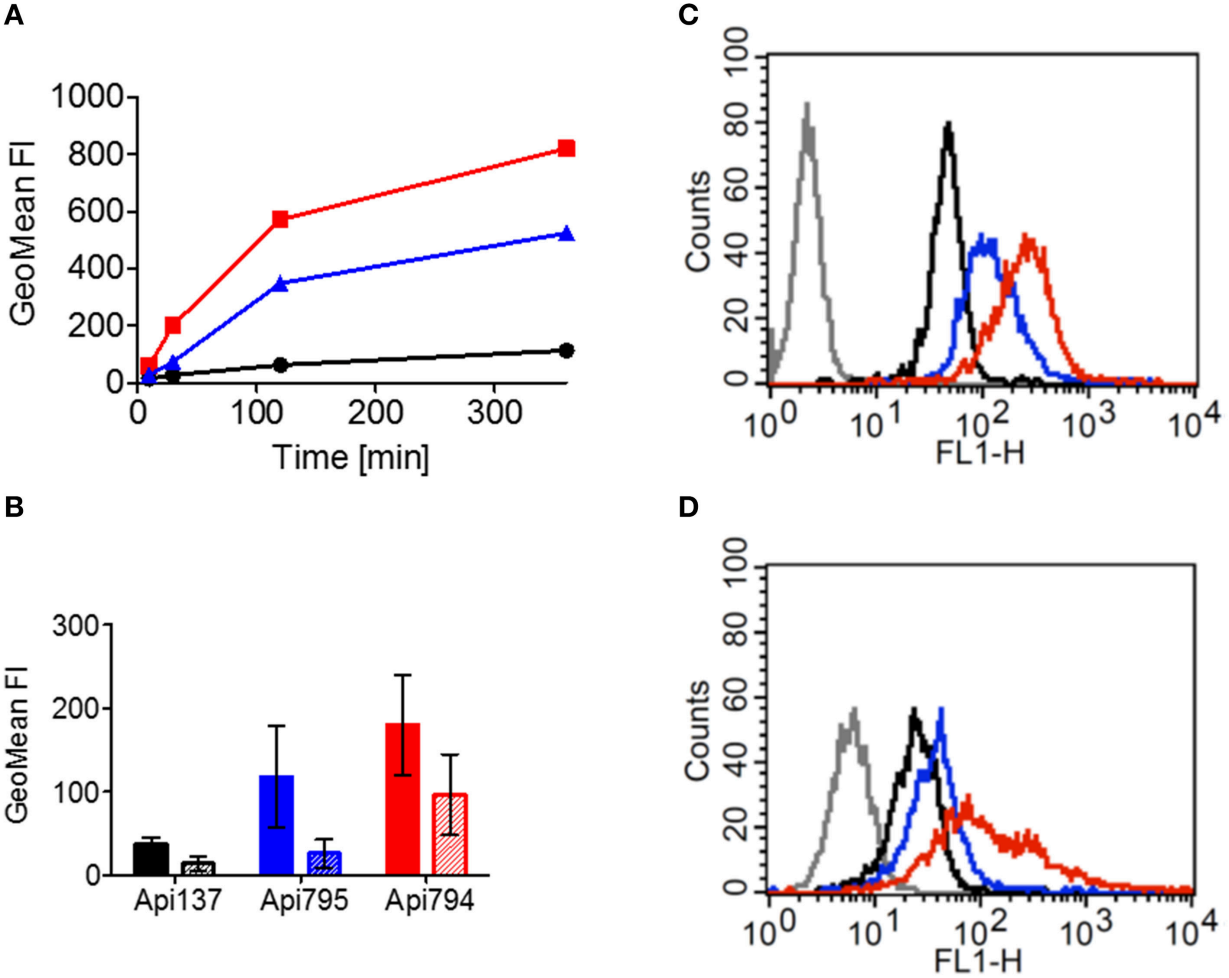
**Uptake of Cf-labeled Api137 (black), Api795 (blue), and Api794 (red) in HeLa cells determined by flow cytometry**. **(A)** HeLa cells were incubated for the indicated times with Api137, Api795, or Api794 (40 μmol/L). **(B)** HeLa cells were treated for 45 min without (filled bars) or with dynasore (0.2 mmol/L, striped bars) before they were treated with a peptide (40 μmol/L) for 30 min. Cells not incubated with a peptide were used as controls (gray in histograms) without **(C)** or with dynasore **(D)**.

Reportedly, 5(6)-carboxyfluorescein-labeled Api137 does not enter mammalian cells (Hansen et al., 2012), which was confirmed here by incubating HeLa cells with Cf-Api137 for 30 min and studying the uptake by laser scanning microscopy, where only very light fluorescence signals were detected (Figure 6A, Figure S7). The fluorescence of Cf-Api794 and Cf-Api795 was located mostly in vesicles and for Cf-Api794 additionally in the cytosol (Figure 6A, Figure S7). Flow cytometry revealed that all three labeled peptides were time dependently internalized (Figure 7A). Relative to the fluorescence intensity of Cf-Api794 after 6 h, the uptake of Cf-Api137 was only 14 and 64% for Cf-Api795. The eFluor660 stain for dead cells confirmed that at least 85% of peptide treated cells were alive revealing that the peptides did not lead to augmented cell death during the experiment (Table S4).

When endocytosis of HeLa cells was inhibited with dynasore for 45 min before addition of the peptides, Cf-Api794 and Cf-Api795 were not detected in vesicles by confocal laser scanning microscopy (Figure 6B, Figure S7). However, Cf-Api794 was still present in the cytosol. Flow cytometry analysis of cells treated with dynasore and Cf-Api795 showed a histogram similar to Cf-Api137 (Figures 7C,D) clearly indicating an unspecific background fluorescence without endocytosis. Cells treated with Cf-Api794 in the presence of dynasore showed a broad distribution of fluorescence intensities ranging from background fluorescence (overlapping with water control) for a few cells to 3000 fluorescence intensity units, which was more than 10-fold higher than observed for the maximum of Cf-Api795. Compared to the vehicle control, the GeoMean fluorescence dropped in the presence of dynasore by 78% for Cf-Api795 and only by 47% for Cf-Api794 (Figure 7B). The already weak fluorescence intensity of Cf-Api137 was diminished by 62%. The significantly lower fluorescence uptake of Api795 indicated that the uptake of this peptide relies mostly on endocytosis. In contrast, Api794 is additionally internalized independent of endocytosis into the cytosol.

**Figure 8:**
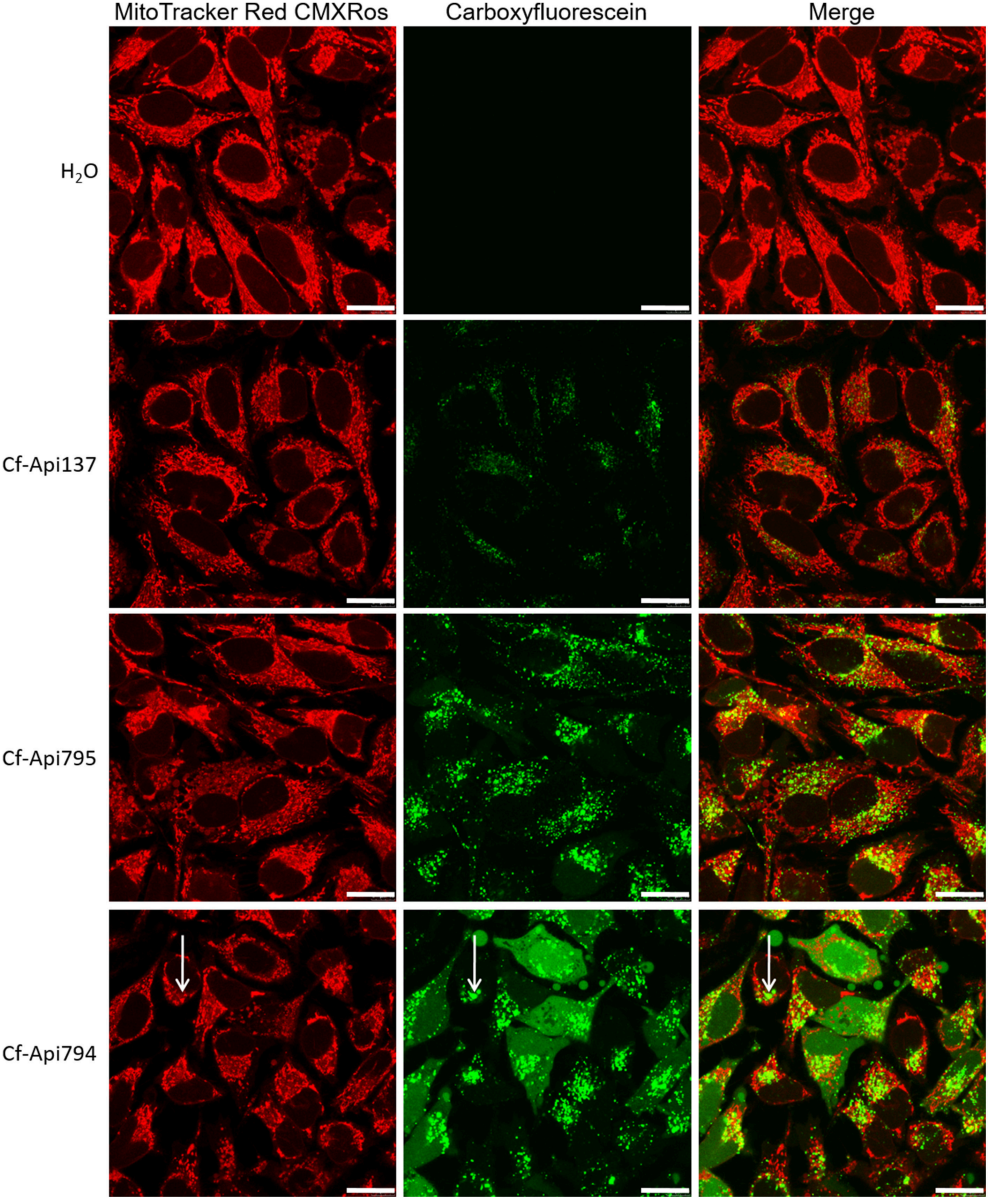
**Localization of the peptides in HeLa cells determined by confocal microscopy**. Cells were incubated with the indicated peptide (40 μmol/L) and MitoTracker Red CMXRos (0.1 μmol/L) for 6 h. Bars refer to 20 μm. Arrows mark the slight background fluorescence of the mitochondrial stain and the high fluorescence of the Cf-labeled peptide, which overlap (unspecific colocalization).

When HeLa cells were incubated for a long period of 6 h with MitoTracker Red CMXRos and fluorescence-labeled peptides, Cf-Api137, Cf-Api794, and Cf-Api795 were visible in vesicles and Cf-Api794 additionally in the cytosol (Figure 8). Most vesicles were asymmetrically distributed around the nucleus. In these areas the green and red fluorescence corresponding to peptide and the MitoTracker, respectively, seemed to be partly overlapped (shown as yellow in the merged images, Figure 8, right). As the colocalized fluorescence was observed for all three partially or non-toxic peptides and appeared only in small areas of the mitochondrial network, it was most likely an unspecific overlap indicating that apidaecin analogs do not interact with the mitochondrial membrane system.

## Discussion

During evolution, bacteria and the immune system of the corresponding hosts have developed various strategies to overcome each other. While pathogenic bacteria have developed strategies to circumvent hosts' immune systems, humans invented a number of different antibiotics during the last century to treat bacterial infections. However, bacteria have caught up in recent decades and are now capable of overcoming antibiotic therapies and thus represent a major health threat especially in immunocompromised persons. The lack of therapeutic options and the low number of antibiotics in clinical phases demand new substances relying on novel mechanisms and targets. In particular, antibiotics against *P. aeruginosa* are urgently needed owing to its versatile resistance mechanisms.

PrAMPs enter bacteria by different mechanisms and inhibit at least the bacterial ribosome and chaperone DnaK, i.e. protein translation and protein folding. The large contact area between a PrAMP and the targeted protein together with their efficacy as part of innate immunity in insects (and mammals) proven for several millions of years indicate a very low probability that bacteria can develop efficient resistances against them.

Api137 and the recently developed derivatives Api755 and Api760 are all active against *P. aeruginosa in vitro*, but only for low nutrient conditions. This clearly indicates that apidaecin analogs are active and thus structural changes could further improve the activity under high salt and nutrient conditions. It must be stressed that MIC values are determined under standardized but artificial conditions providing ideal growth conditions for bacteria lacking any other substances interfering with bacterial growth besides the tested compound. In contrast, a host (i.e. patient) does not provide an ideal environment for pathogens and uses a combination of different measures to suppress bacterial growth and finally to kill the pathogens, i.e. synergistic strategies that include AMPs indicating that AMPs might be more active *in vivo* than generally assumed from their MIC values.

In continuation of a previous study (Bluhm et al., 2015), we could increase the activity of apidaecins by elongating the sequences with two-residue motifs containing a basic (Orn) and a hydrophobic amino acid (Trp or Ile) at the N-terminus. The most promising new analogs Api794 and Api795 were up to eight-fold more active than Api755 and Api760 against *P. aeruginosa* even in undiluted MHB, which has not been reported for apidaecin analogs or other short, insect-derived PrAMPs. The basic residues increase the positive net charge of the peptide and most likely allow stronger electrostatic interactions with the negatively charged bacterial surface. The hydrophobic residues, especially Trp, support membrane penetration in general (de Planque et al., 2003; Rekdal et al., 2012) and maybe in particular that of the inner membrane of *P. aeruginosa* that lacks transporter SbmA required for active transport in Gram-negative bacteria (Mattiuzzo et al., 2007; Krizsan et al., 2015a). On the contrary, the slight activity loss observed for both *E. coli* and *K. pneumoniae* might be explained by a less efficient transport by SbmA due to the longer N-terminal sequence or the hydrophobic and basic residues, although the binding site of PrAMPs to SbmA is unknown. The presumably more efficient interaction with bacterial membranes was supported by QCM data for Api794 and Api795 that both showed a transmembrane insertion similar to Api137, but triggered some additional membrane restructuring. Api795 insertion generated a thicker membrane and a more rigid layer. The effect on the membrane was even stronger for Api794 that clearly restructured the surface in addition to its transmembrane insertion.

Electron microscopy studies on bacterial cells showed different morphologic changes induced by Api137, Api794, and Api795 highlighting again that Trp and Ile affect or alter the mechanism of bacterial killing. Although, TEM shows morphological changes that might be related to the mode of action of an antibiotic or represent only later (secondary) effects of starving or dying cells, it still allows deducing important observations that can support or partially disprove mechanistic studies.

Importantly and in agreement with QCM, the peptides did not lyse membranes at their MIC. Even at the highest concentration (4 × MIC) membranes of *E. coli* and *P. aeruginosa* were only perturbed and membranous parts were released. However, no leakage of cytosolic material was observed, contrary to reports on for example temporin L and human HE2 peptide (Mangoni et al., 2004; Yenugu et al., 2004). This strongly suggests that a pure lytic mechanism, where peptides solely act on the bacterial membranes, appears unlikely.

An interesting characteristic was the formation of small vesicles or protrusions at the outer membrane of *P. aeruginosa* (sub-MIC Api794) and *E. coli* (sub-MIC Api137 and MIC Api 794). Morphologically comparable vesicle formation was described for *E. coli* treated with Gramicidin S, a lytic peptide that targets exclusively the bacterial membrane (Hartmann et al., 2010). However, other non-proteinaceous toxic compounds, such as alkanols, also induce vesicle release (Baumgarten et al., 2012), which is considered a general stress response in Gram-negative bacteria (McBroom and Kuehn, 2007). Overexpression of (misfolded) proteins in the periplasmic space for example, triggered similar bacterial vesicle formations. The vesicles contained outer membrane and periplasmic components and thus might remove toxic compounds from cells. It is easily envisioned that Gram-negative bacteria try similarly to remove membrane interacting peptides, even if the membrane is not the final target of these peptides.

The observed relocalization of ribosomes and DNA is comparable to the effect observed in a recent study of pleurocidin and to a lower extent magainin II on *E. coli* (Kozlowska et al., 2014). At concentrations below their MIC a nuclear condensation is described for both peptides, morphologically similar to our observations for apidaecin analogs. While pleurocidin was thought to be a pore-forming peptide with the cytosolic membrane as primary target (Yoshida et al., 2001), other studies have shown that it is actually translocated over the membrane and interferes with several macromolecular processes, including protein and RNA synthesis (Patrzykat et al., 2002). Since intracellular killing is a well described mode of action for natural PrAMPs including apidaecin analogs (Otvos, 2002; Scocchi et al., 2011; Krizsan et al., 2014, 2015b), it would be tempting to correlate the observed intracellular morphological changes to intracellular activity of AMPs, such as apidaecin analogs and pleurocidin. However, this requires a comparative study involving more peptides with known antibacterial mechanisms to validate this correlation.

Overall, the current TEM results confirmed our previous studies that Api795 and Api794 are most active in antibacterial killing against *P. aeruginosa*, but are less efficient against *E. coli*. For therapeutic applications, however, the activity loss against *E. coli* can be tolerated, as the dose will be determined by the least susceptible pathogen, which is at least *in vitro* still *P. aeruginosa*.

The stronger effects on the membrane may explain the lower MICs of both Api794 and Api795, but they are unfortunately not limited to prokaryotic membranes and increased also adverse effects of Api794 on mammalian cell cultures. In this respect, Api795 containing Ile instead of Trp residues appears to act by favorably balanced membrane effects that increase the activity against *P. aeruginosa* without initiating adverse effects on mammalian cell lines, as indicated by cell viability and LDH release. The lower tolerance of Api794 compared to Api795 was supported by flow cytometry and laser scanning microscopy indicating different uptake mechanisms of the fluorescence-labeled derivatives in mammalian cells. Cf-Api795 entered HeLa cells in relatively large quantities by endocytosis, but still 30% less efficient than Cf-Api794. After inhibition of endocytosis, Cf-Api794 remained localized in the cytoplasm. This supports the hypothesis that neither Api794 nor Api795 lead to membrane permeabilization, which was additionally confirmed by the lack of LDH release in cardiomyocytes. The observed endocytosis and the colocalization of Cf-Api794 or -Api795 containing vesicles did most likely not affect cell viability.

## Conclusion

PrAMPs including Api137 have been successfully evaluated in different murine *Enterobacteriaceae* infections models giving reasonable hope that they are highly active and well tolerated providing a large therapeutic window. Here, we could develop new analogs with much more activity against *P. aeruginosa* under full medium conditions. Among the new derivatives Api795 appears to be the most promising, as it is highly active against *E. coli, K. pneumoniae*, and *P. aeruginosa*, non-hemolytic, and only slightly toxic against mammalian cells even at high concentrations. Mechanistically, Api795 shows a transmembrane insertion into bacterial mimic membranes and initiates a structural change leading to a thicker and more rigid membrane layer. The TEM result demonstrated that all three peptides, despite their high sequence homology, have very different effects on the morphology of bacteria, and that these observable changes are very different for *P. aeruginosa* and *E. coli*.

## Author contributions

MB: peptide synthesis, MIC determination, cytotoxicity assays, FACS assays, cell preparation for confocal fluorescence microscopy, parts of Quartz crystal microbalance experiments, manuscript preparation. VS: TEM, manuscript preparation. IS: confocal fluorescence microscopy. SP: quartz crystal microbalance experiments. TG: technical support FACS experiments. DK: manuscript preparation, discussions. PS: confocal fluorescence microscopy. LM: data evaluation quartz crystal microbalance experiments, manuscript preparation. EV: data evaluation TEM, manuscript preparation. RH: Project supervision, manuscript preparation.

### Conflict of interest statement

RH is cofounder of AMP-Therapeutics GmbH (Leipzig, Germany) and DK was part time coworker of AMP-Therapeutics GmbH. All other authors declare that the research was conducted in the absence of any commercial or financial relationships that could be construed as a potential conflict of interest.
