# Supplementary material for: N-Terminal Ile-Orn- and Trp-Orn-Motif Repeats Enhance Membrane Interaction and Increase the Antimicrobial Activity of Apidaecins against Pseudomonas aeruginosa
Source: Front Cell Dev Biol. 2016 May 10;4:39. doi: 10.3389/fcell.2016.00039 (PMC4861708; doi:10.3389/fcell.2016.00039)

Supplement

N-Terminal Ile-Orn- and Trp-Orn-Motif Repeats Enhance Membrane Interaction and Increase the Antimicrobial Activity of Apidaecins against *Pseudomonas aeruginosa*

Martina E.C. Bluhm[1,2], Viktoria A.F. Schneider[3], Ingo Schäfer[2,4], Stefania Piantavigna[5], Tina Goldbach[1,2], Daniel Knappe[1,2], Peter Seibel[2,4], Lisandra L. Martin[5], Edwin J.A. Veldhuizen[3] and Ralf Hoffmann[1,2]

[1]Institute of Bioanalytical Chemistry, Faculty of Chemistry and Mineralogy, Universität Leipzig, Leipzig, Germany

[2]Center for Biotechnology and Biomedicine (BBZ), Universität Leipzig, Leipzig, Germany

[3]Department of Infectious Diseases and Immunology, Division of Molecular Host Defence, Faculty of Veterinary Medicine, Utrecht University, Utrecht, the Netherlands

[4]Molecular Cell Therapy, Faculty of Medicine, Universität Leipzig, Leipzig, Germany

[5]School of Chemistry, Monash University, Victoria, Australia

Table of contents

Table S1: MIC-values of Api137, Api794, and Api795 determined against *P. aeruginosa* DSM 1117 and *E. coli* DSM 1103 using an inoculum of 5 x 10^8 cells/mL……………………………………………………………………..…………………….**2**
Table S2: Quantification of morphological changes of *P. aeruginosa* DSM 1117 cells….......**2**
Table S3: Quantification of morphological changes of *E. coli* DSM 1103 cells……………...**3**
Table S4: Survival rates of HeLa cells after incubating them with Cf-labeled apidaecin peptides……………………………………………………………………………….......**3**
Figure S1: RP-HPLC and MALDI-TOF-MS of all purified apidaecin analogs used in this study……………………………………………………………………………………...**4**
Figure S2: RP-HPLC and MALDI-TOF-MS of all purified 5(6)-carboxyfluorescein labeled apidaecin peptides used in this study……………………………………………………**6**
Figure S3: Electron microscopy images with higher magnification of *P. aeruginosa* ………………………………………………………………………………….......................**7**
Figure S4: Electron microscopy images with higher magnification of *E. coli*….. …………………………………………………………………………….........................**8**

Figure S5: Electron microscopy images of bacteria incubated with high peptide concentrations...**9**
Figure S6: RP-Chromatograms of LL-37, Api137, Api794, and Api795 before and after incubation with *P. aeruginosa* elastase...**9**
Figure S7: Confocal microscopy images with higher magnification of HeLa cells treated with or without dynasore prior to incubation with Cf-labeled apidaecin peptides.....................**10**

Table S1: MIC-values in µg/mL of Api137, Api794, and Api795 determined against *P. aeruginosa* DSM 1117 and *E. coli* DSM 1103 in 50% MHB and 33% TSB, respectively, using an inoculum of 5 x 10^8 cells/mL corresponding to the conditions applied for electron microscopy.

	P. aeruginosa DSM1117			*E. coli* DSM 1103		
Peptide	0.25x MIC	MIC	4x MIC	0.25x MIC	MIC	4x MIC
Api137	32	128	512	8	32	128
Api794	16	64	256	16	64	256
Api795	4	16	64	8	32	128

Table S2: Quantification of morphological changes of *P. aeruginosa* DSM 1117 cells [%].

	Conc. [µg/mL]	Membrane i	Membrane b	Cells r	Cells spongy	DNA/ribosomes relocalization -	+	++	+++	Ribosome clustering -	+	++	+++	Vesicle release	dissociated Fragments -	+	++	LV
**Water**	-	100	0	0	0	90	10	0	0	87	13	0	0	13	87	13	0	0
**Api137**	32	100	0	0	0	40	43	17	0	37	63	0	0	43	87	13	0	0
	128	100	0	0	0	47	37	17	0	43	57	0	0	47	87	10	3	0
	512	100	0	0	0	47	40	13	0	70	30	0	0	37	97	0	3	0
**Api795**	4	100	0	0	0	40	37	23	0	30	70	0	0	67	93	3	3	0
	16	100	0	0	0	50	40	10	0	70	30	0	0	13	100	0	0	0
	64	90	10	3	0	10	10	67	13	10	77	7	7	50	97	0	3	0
	512	20	80	63	10	0	0	0	100	0	0	7	93	7	87	0	13	0
**Api794**	16	100	0	0	0	40	37	23	0	20	80	0	0	87	77	10	13	0
	64	20	80	47	0	3	3	10	83	3	13	10	73	20	17	20	63	30
	256	34	67	67	80	0	0	0	100	0	0	0	100	3	7	13	80	93
	512	17	13	17	87	0	0	0	100	0	0	7	93	0	40	0	60	97

i, intact; b, broken; r, ruptured; DNA/ribosome relocalization and ribosome clustering: -, no; +, starting; ++ relocalized/clustered; +++, extremely relocalized/clustered; fragments: -, no; +, small and thin; ++, long and thin; LV, large vesicles

Tab. S3: Quantification of morphological changes of *E. coli* DSM 1103 cells [%].

	Conc. [µg/mL]	Membrane i	Membrane w	Cell r	DNA/Ribosomes relocalized	Vesicle release weak	Vesicle release strong	Fragments -	Fragments +	Fragments ++	Fragments +++	Fragments LV
**water**	-	13	87	0	0	13	-	33	63	3	0	0
**Api137**	8	100	0	0	17	-	100	43	30	27	0	0
	32	97	3	0	30	-	100	37	40	23	0	0
	128	4	96	0	0	35	-	4	54	42	0	0
	512	32	68	0	0	50	-	11	61	29	0	0
**Api795**	8	3	97	0	0	30	-	13	63	23	0	0
	32	7	93	0	0	43	-	33	57	10	0	0
	128	17	83	0	0	47	-	17	53	30	0	0
	512	33	67	0	30	60	-	0	63	37	0	0
**Api794**	16	33	67	0	0	-	100	37	40	23	0	0
	64	100	0	0	0	-	97	17	27	33	23	3
	256	3	97	0	0	-	97	17	43	37	3	93
	512	93	7	3	30	-	93	17	37	30	17	97

i, intact; w, wrinkled; r, ruptured; fragments: -, no; +, small and thin; ++, long and thin; +++ long and thick; LV, large vesicles

Table S4: Survival rates of HeLa cells after incubating them with Cf-labeled apidaecin peptides for 10 min or 6 h. The fluorescence corresponding to 5(6)-carboxyfluorescein and eFluor660 to stain dead cells were measured by flow cytometry. Dead cells were excluded from the population. Experiments were performed twice (Exp1/Exp2).

Time	Water	Cf-Api137	Cf-Api794	Cf-Api795
10 min	90% / 87%	89% / 85%	91% / 85%	90% / 85%
6 h	93% / 86%	93% / 91%	85% / 84%	93% / 86%

Figure S1: RP-HPLC and MALDI-TOF-MS of all purified apidaecin analogs used in this study. RP-HPLC relied on a Jupiter C_{18}-column and a linear aqueous acetonitrile gradient from 21-33% (v/v) acetonitrile in 30 min in the presence of TFA (0.1% v/v; left). Mass spectra were recorded on a Synapt G2Si mass spectrometer equipped with a MALDI-source using CHHA as matrix (right).

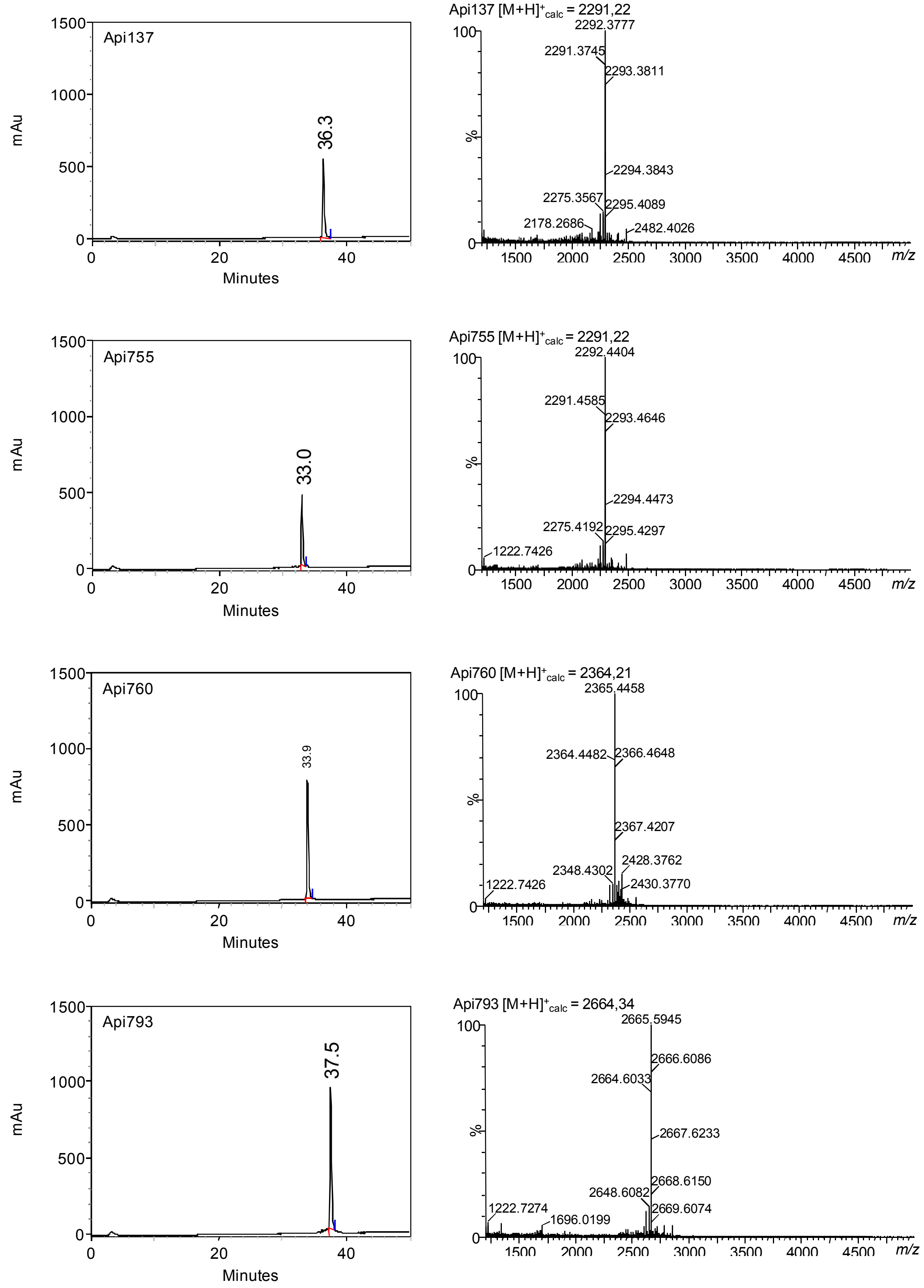

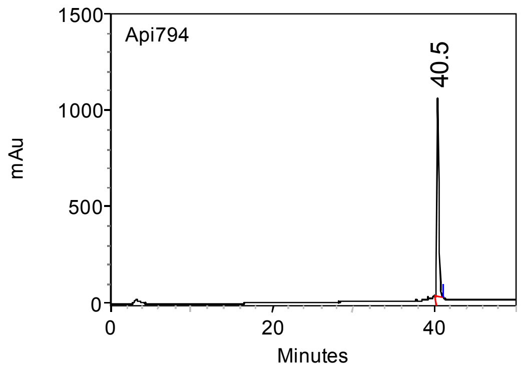
Api794
40.5
mAu
1500
1000
500
0
0
20
40
Minutes

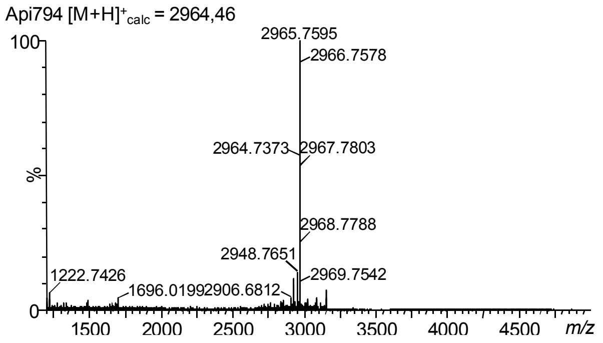
Api794 [M+H]+calc = 2964,46
2965.7595
2966.7578
2964.7373
2967.7803
2968.7788
2948.7651
2969.7542
1222.7426
1696.0199
2906.6812
%
100
0
1500
2000
2500
3000
3500
4000
4500
m/z

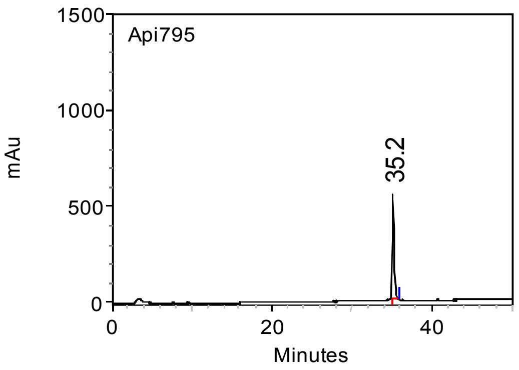
Api795
35.2
mAu
1500
1000
500
0
0
20
40
Minutes

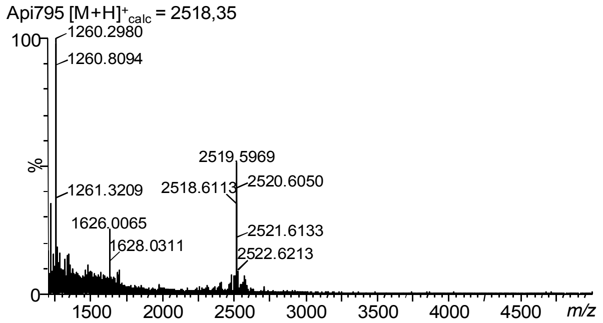
Api795 [M+H]+calc = 2518,35
1260.2980
1260.8094
1261.3209
1626.0065
1628.0311
2519.5969
2518.6113
2520.6050
2521.6133
2522.6213
%
100
0
1500
2000
2500
3000
3500
4000
4500
m/z

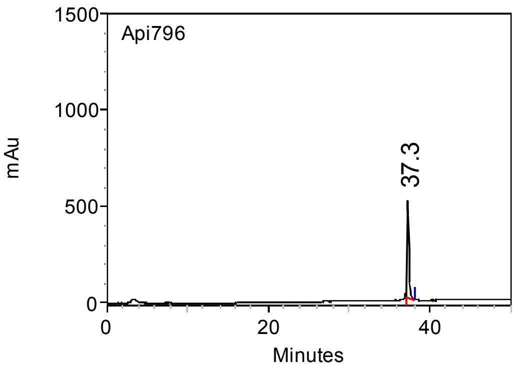
Api796
37.3
mAu
1500
1000
500
0
0
20
40
Minutes

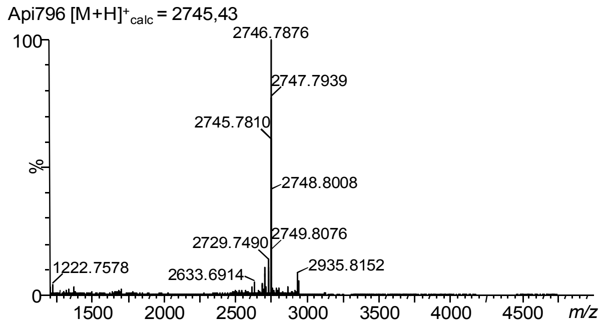
Api796 [M+H]+calc = 2745,43
2746.7876
2747.7939
2745.7810
2748.8008
2729.7490
2749.8076
1222.7578
2633.6914
2935.8152
%
100
0
1500
2000
2500
3000
3500
4000
4500
m/z

Figure S2: RP-HPLC and MALDI-TOF-MS of all purified 5(6)-carboxyfluorescein labeled apidaecin peptides used in this study. RP-HPLC relied on a Jupiter C_{18}-column and a linear aqueous acetonitrile gradient from 31.5-49.5% (v/v) acetonitrile in 30 min in the presence of TFA (0.1% v/v; left). Mass spectra were recorded on a Synapt G2Si mass spectrometer equipped with a MALDI-source using CHHA as matrix (right).

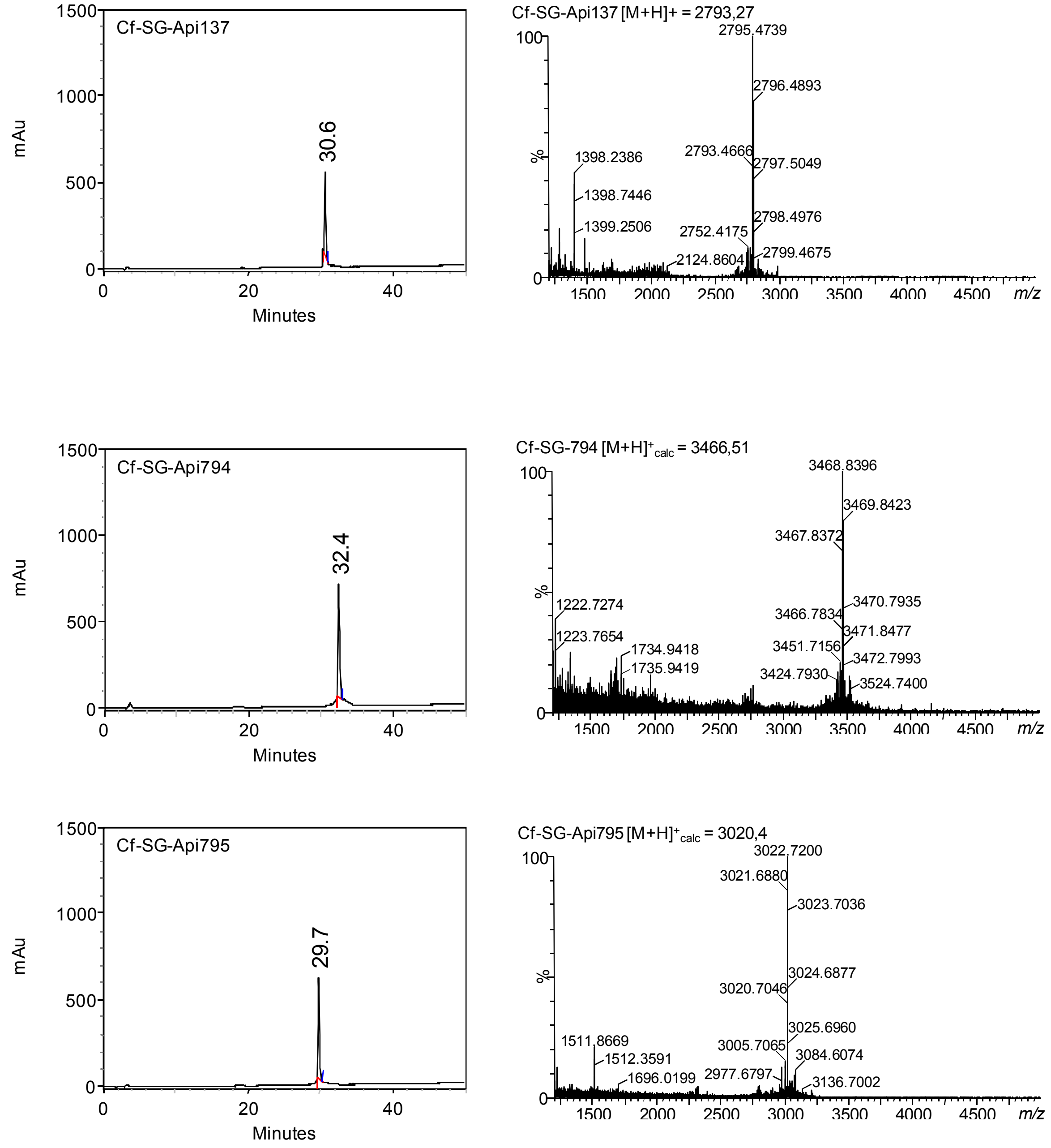

Figure S3: Electron microscopy images of *P. aeruginosa* DSM 1117 incubated with Api137 (A), Api795 (B), and Api794 (C) at concentrations corresponding to 0.25x MIC, MIC, and 4x MIC for 1 h using cell densities of 5 x 10^8 CFU/mL. Black bars represent 200 nm.

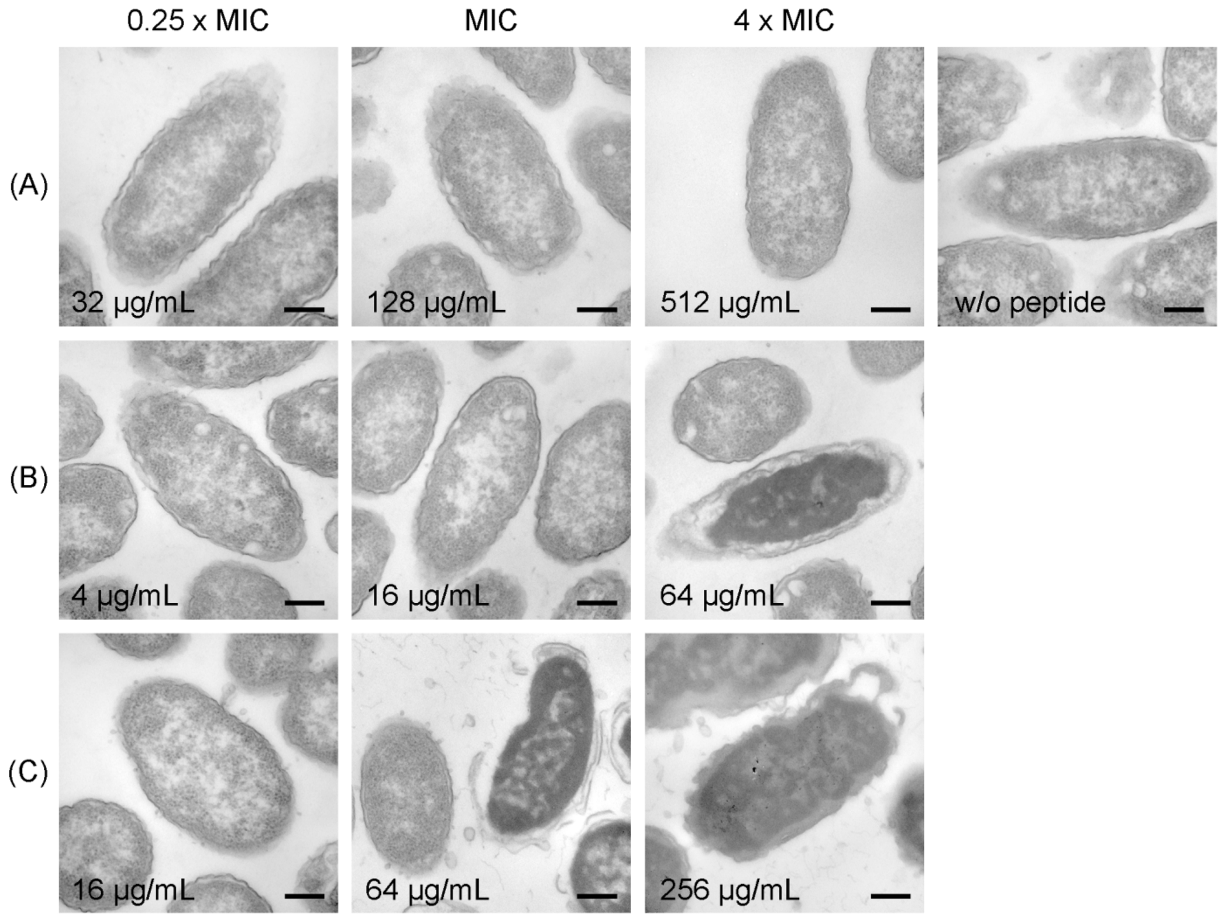

Figure S4: Electron microscopy images of *E. coli* DSM 1103 incubated with Api137 (A), Api795 (B), and Api794 (C) at concentrations corresponding to 0.25x MIC, MIC, and 4x MIC for 1 h using cell densities of 5 x 10^8 CFU/mL. Black bars represent 200 nm.

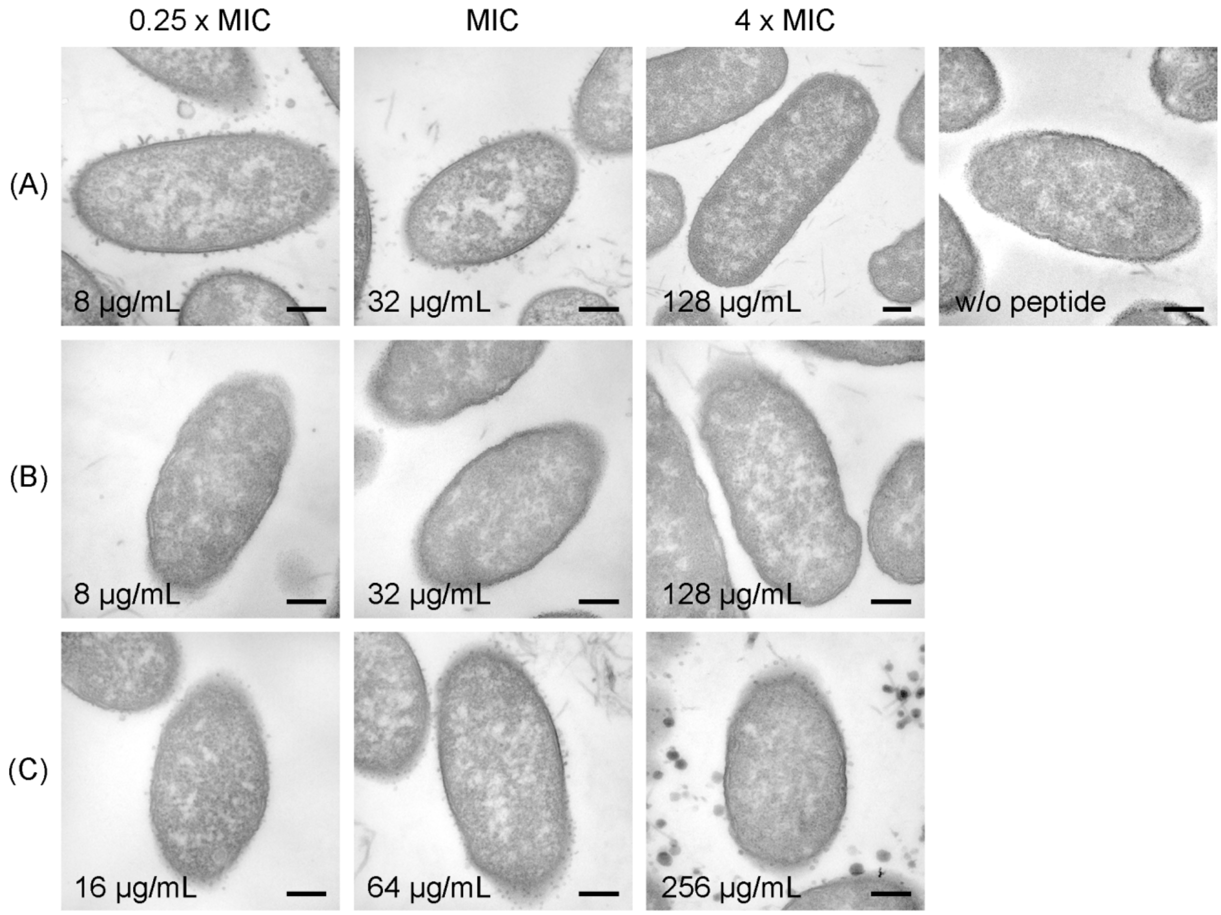

Figure S5: Electron microscopy images of *P. aeruginos*a DSM 1117 (A) and *E. coli* DSM 1103 (B) incubated with the indicated apidaecin analogs (512 µg/mL) for 1 h using cell densities of 5 x 10^8 CFU/mL. Black bars refer to 500 nm.

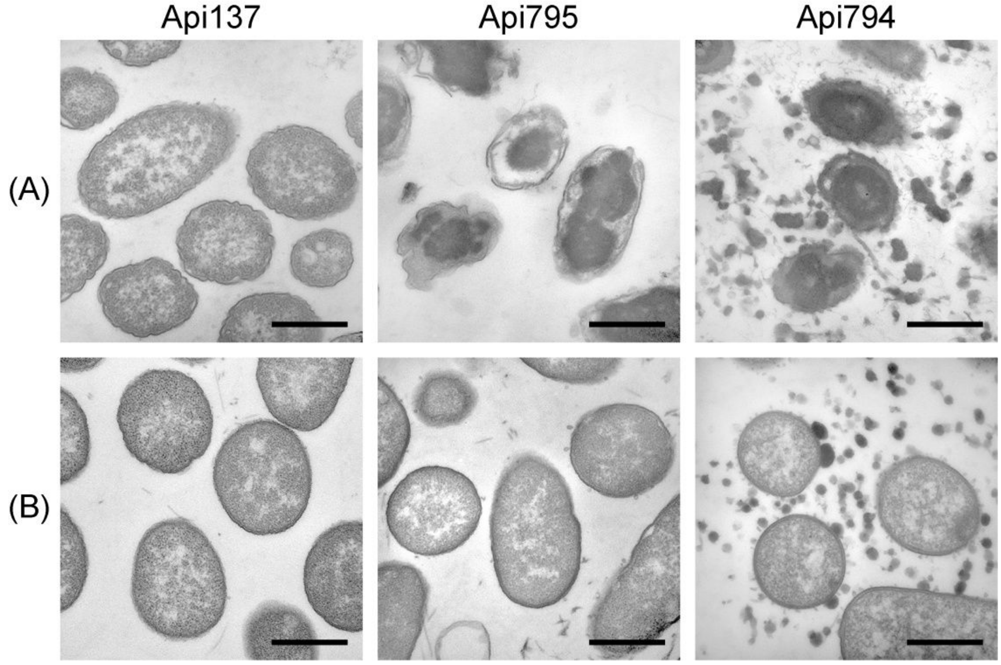

Figure S6: RP-chromatograms of LL-37, Api137, Api794, and Api795 before and after incubation at a concentration of 0.5 g/L with *P. aeruginosa* elastase (12 units/mL) in Tris-HCl buffer (pH 8.3) at 37 °C.

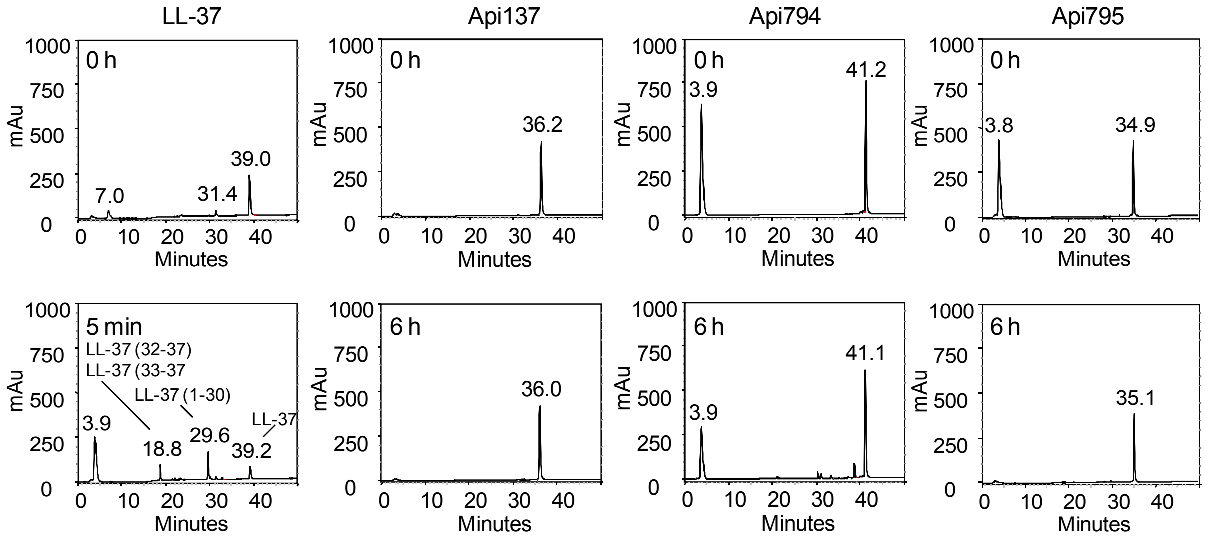

Figure S7: Confocal microscopy images of HeLa cells incubated with Cf-labeled apidaecin peptides (green). Cells were treated without (A) or with dynasore (B); 0.2 mmol/L) for 45 min prior to peptide treatment (40 µmol/L, 30 min). The cells' nuclei were visualized by the use of Hoechst 33324 (blue). Bars refer to 20 µm. Arrows indicate examples of areas with endosomes (white) and stained cytosol (red).

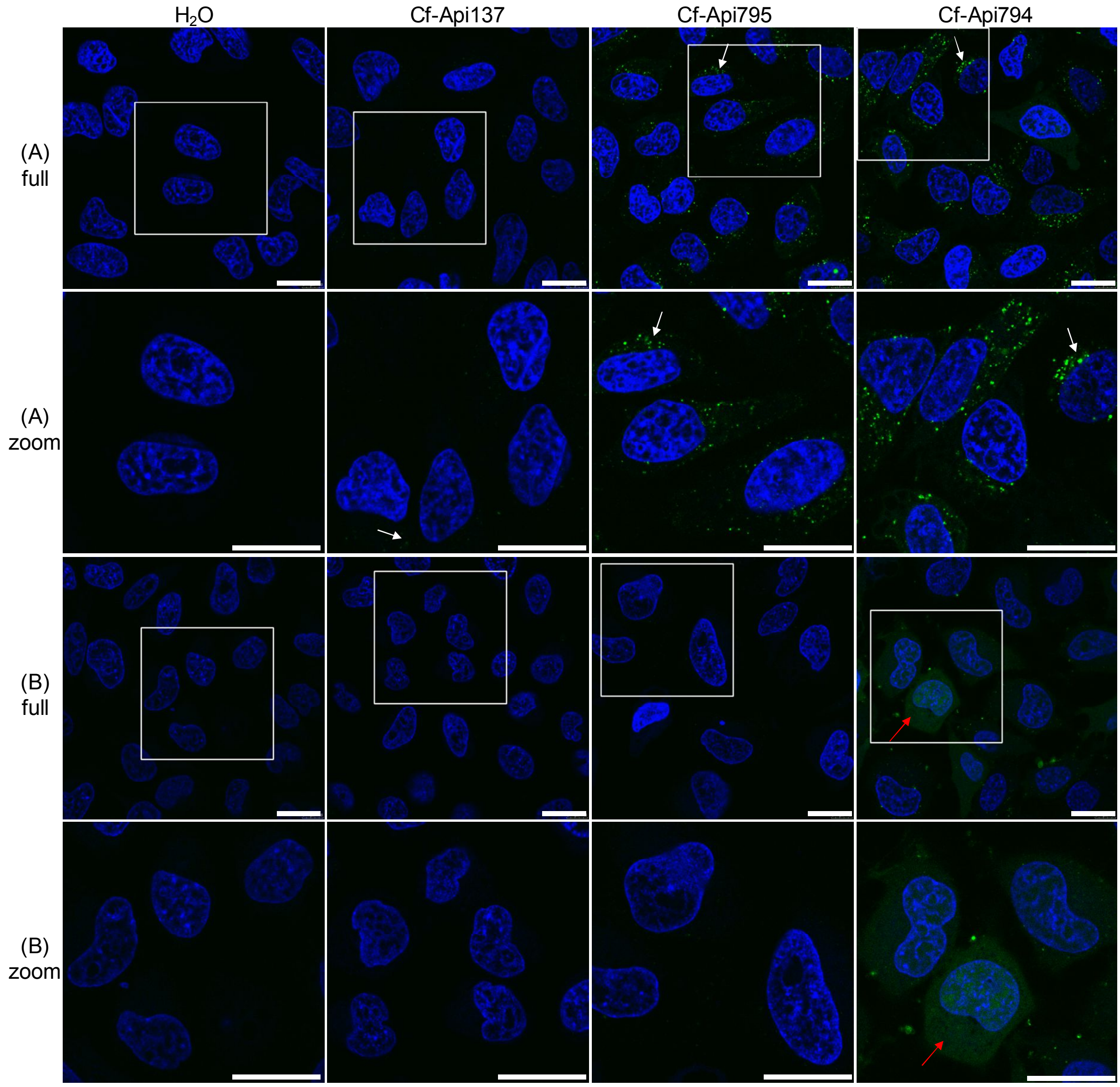

Supplement: Supplementary file 1 [file Presentation1.pdf]
